# Plant community composition steers grassland vegetation via soil legacy effects

**DOI:** 10.1111/ele.13497

**Published:** 2020-04-07

**Authors:** Robin Heinen, S. Emilia Hannula, Jonathan R. De Long, Martine Huberty, Renske Jongen, Anna Kielak, Katja Steinauer, Feng Zhu, T. Martijn Bezemer

**Affiliations:** ^1^ Department of Terrestrial Ecology Netherlands Institute of Ecology P.O. Box 50 6700 AB Wageningen The Netherlands; ^2^ Institute of Biology Section Plant Ecology and Phytochemistry Leiden University P.O. Box 9505 2300 RA Leiden The Netherlands; ^3^ Key Laboratory of Agricultural Water Resources, Hebei Key Laboratory of Soil Ecology Center for Agricultural Resources Research Institute of Genetic and Developmental Biology The Chinese Academy of Sciences 286 Huaizhong Road 050021 Shijiazhuang Hebei China; ^4^ Present address: Lehrstuhl für Terrestrische Ökologie Landnutzung und Umwelt Technische Universität München Wissenschaftszentrum Weihenstephan für Ernährung Hans‐Carl‐von‐Carlowitz‐Platz 2 D‐85354 Freising Germany

**Keywords:** Field experiment, grassland, pathogens, plant-soil feedback, soil bacteria, soil fungi, soil legacy effects, soil microbiome

## Abstract

Soil legacy effects are commonly highlighted as drivers of plant community dynamics and species co‐existence. However, experimental evidence for soil legacy effects of conditioning plant communities on responding plant communities under natural conditions is lacking. We conditioned 192 grassland plots using six different plant communities with different ratios of grasses and forbs and for different durations. Soil microbial legacies were evident for soil fungi, but not for soil bacteria, while soil abiotic parameters did not significantly change in response to conditioning. The soil legacies affected the composition of the succeeding vegetation. Plant communities with different ratios of grasses and forbs left soil legacies that negatively affected succeeding plants of the same functional type. We conclude that fungal‐mediated soil legacy effects play a significant role in vegetation assembly of natural plant communities.

## Graphical abstract

Under natural conditions, plant communities with different composition drive microbe‐mediated soil legacy effects on subsequent vegetation. Specifically, the abundance of forbs and grasses in vegetation can result in soil communities that negatively affect plants belonging to the same respective group. This study shows that plant–soil feedbacks occur in the field and are – in part – driven by pathogenic soil fungi.

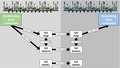

## Introduction

Plants and soil organisms are interdependent and the microbiome in the soil is shaped by the plants that grow in the soil (Phillipot *et al.* 2013; Bardgett & Van der Putten 2014). This microbial signature can remain as a legacy in the soil after the plant is gone, and in turn affect other plants growing later in the same soil (Kulmatiski *et al.* 2008; Van der Putten *et al.* 2013; Teste *et al.* 2017; Eppinga *et al.* 2018). It is often speculated that soil legacy effects created by plants play an important role in regulating plant community dynamics and plant coexistence (Lekberg *et al.* 2018; Semchenko *et al.* 2019). It was recently shown that inoculation of soils with biotic legacies can change plant community development under natural conditions (Wubs *et al.* 2016; Wubs *et al.* 2019). However, experimental evidence for soil legacy effects of plant communities with different characteristics on responding plant communities in natural systems is lacking (Reynolds *et al.* 2003; Ehrenfeld *et al.* 2005; Van der Putten *et al.* 2013).

Herbaceous grassland plant species such as grasses (monocots) and forbs (dicots) differ fundamentally in root architecture (Craine *et al.* 2001, 2002; Ravenek *et al.* 2016), water and nutrient acquisition (Tjoelker *et al.* 2005; Ravenek *et al.* 2016), and in defense (Latz *et al.* 2015, 2016; Zhang, Van der Putten & Veen 2016). These differences between plant functional types can modulate soil communities (Kos *et al.* 2015; Latz *et al.* 2015; Zhang, Van der Putten & Veen 2016), leaving soil legacy effects that affect subsequent plant growth (Wubs & Bezemer 2018; Heinen *et al.* 2018; Heinen, Biere & Bezemer, 2019). Generally, grass and forb species exhibit negative conspecific soil legacy effects (Kulmatiski *et al.,* 2008), which is often explained by the accumulation of specialised pathogens (Van der Putten *et al.,* 2013). However, growing in conspecific soil can also lead to positive effects through the accumulation of mutualists in the soil (Morrien *et al.,* 2017; Hannula *et al.* 2017; Teste *et al.* 2017). In pot experiments, grasses often have increased performance on soils conditioned by forb species and *vice versa* (Petermann *et al.* 2008; De Kroon *et al.* 2012; Wubs & Bezemer 2018). As plant species‐specific communities of soil organisms develop around the roots of plants, soil legacies may become stronger over time (Diez *et al.* 2010). While it has been shown that individual plants in the field influence their local soil community (De Rooij‐Van der Goes, Peters & Van der Putten 1998; Bezemer *et al.* 2006; Casper & Castelli 2007; Van de Voorde *et al.* 2011; Hannula *et al.* 2019a,b), how different plant communities drive soil legacies in the field and how this affects the establishment of responding mixed plant communities in these soils is not known (Ehrenfeld *et al.* 2005; Kardol *et al.* 2007; Van der Putten *et al.* 2013).

We grew six different plant communities in a temperate grassland. Each plant community consisted of a combination of grass and/or non‐leguminous forb species (hereafter: forbs) which were grown in different ratios (0:100; 25:75; 75:25 or 100:0% forb:grass respectively). The (sub)plots were exposed to different durations of conditioning by starting the treatments in two different years. After the conditioning phase of one or two years, all plant communities were removed from the soil, and the same seed mixture of 33 grassland species was sown in each treatment (sub)plot as a responding plant community. In both phases we recorded the abundance of all plant species, soil abiotic characteristics, and soil fungal and bacterial community composition. In the conditioning phase, we expected that plant communities would influence soil abiotic characteristics and soil biotic composition, and we expected that the soil biota would affect the establishment of future plant communities in the responding phase.

We hypothesised that manipulation of the composition of the conditioning plant communities will result in different microbial soil legacies, and specifically in the accumulation of specialised soil pathogens and mutualists such as arbuscular mycorrhizal fungi (AMF). Second, we hypothesised that in the response phase, grasses and forbs would be less abundant in soils that had been dominated by their own functional type in the conditioning phase, due to the accumulation of soil pathogens. Third, we hypothesised that these effects would be stronger in soils with a two‐year legacy than in one‐year legacy soils, due to the gradual development of specific soil microbiomes over time. Lastly, we hypothesised that soil legacy effects would be mediated by microbial changes in the soil, rather than by soil abiotic characteristics.

## Materials and Methods

### Experimental setup

#### Study site

In 2015, the field experiment was set up in a restored grassland site (abandoned from agricultural use in 1996), ‘De Mossel’ (Natuurmonumenten, Ede, The Netherlands, 52°04´ N, 5°45´ E). Soils are holtpodzol, sandy loam (94% sand, 4% silt, 2% clay, *c*. 4% organic matter, 5.2 pH, 2.5 mg kg^−1^ N, 4.0 mg kg^−1^ P, 16.5 mg kg^−1^ K) (Jeffery *et al.* 2017). The native vegetation in this site is dominated by typical grassland species such as *Achillea millefolium* L.*, Jacobaea vulgaris* Gaertn.*, Plantago lanceolata* L. *Taraxacum officinale* Wigg*, Agrostis capillaris* L., *Holcus lanatus* L. *Lolium perenne* L. and *Phleum pratense* L. (Morrien *et al.* 2017). Average daily temperatures in the area are 16.7 °C in summer months and 1.7 °C in winter months. Average monthly precipitation ranges from 48 to 76 mm (www.climate‐data.org).

#### Phase 1: Conditioning phase

The experimental design of the conditioning phase has been described in full detail in De Long *et al. *(2019). In total there were 96 plots of 166 × 250 cm. Each plot was divided into two 83 × 250 cm subplots. A specific seed mixture was allocated to each plot (and hence to the two subplots). The plant species in the mixtures were selected from two separate pools of plant species. Three seed mixtures consisted of random combinations from a pool that contained 12 plant species considered to be faster‐growing plant species (communities 1–3 hereafter) and the three remaining seed mixtures consisted of random combinations from a pool containing 12 species considered to be slower‐growing plant species (communities 4–6; Table S1; De Long *et al.* 2019). Each seed mixture differed from the others but always consisted of three grass species and three forb species (Table S2). To test the effects of plant functional types in the conditioning plant community, each seed mixture was prepared in four different forb:grass ratios (i.e. 0:100%, 25:75%, 75:25% 100:0% forb:grass) so that there were 6 seed mixtures x 4 ratios = 24 unique communities. Each seed mixture was sown in four blocks totaling 96 plots (each consisting of two subplots). The two subplots within each plot were sown in consecutive years, to create one‐year or two‐year legacies, to test whether soil legacy effects would become stronger when the period of conditioning was longer.

In May 2015 (i.e. two‐year legacy treatments), one of the two subplots of each plot was randomly selected. All original vegetation of the subplot was removed using shovels, while the other subplot was left untouched in that year. Removal included the top soil layer of approximately 4 cm, which generally contains the highest density of roots in this grassland system. This was done to remove the most dominant roots of the plants and prevent re‐growth of non‐target plant species. Each stripped subplot was then sown with the seed mixture that was allocated to that plot as described above. In May 2016 (i.e. one‐year legacy treatments), all vegetation was removed (as described above) from the remaining untouched subplot in each of the 96 plots. These subplots were then sown with the seed mixture that was also sown a year earlier in the paired subplot.

#### Phase 2: Responding phase

At the end of the conditioning phase, on 12–16 June 2017, the vegetation was again removed from all subplots using a sod‐cutting machine (IB200, IBEA, Tradate, Italy). All sods were cut to a standard depth of 3 cm. This was done to remove most of the thicker roots and to prevent re‐growth. After cutting, the soil was hand‐shaken from the sods above the subplots, allowing us to keep most of the remaining soil from the sods in the respective (sub)plots. On 20 June 2017, all subplots were sown with the same seed mixture consisting of the all species sown in the conditioning phase plus ten others that occur in the area but not at the site (Bezemer, personal observation; Table S1). Subplots were watered regularly in the first month to assist establishment of the germinating seedlings and then left to develop naturally. Disturbance was minimalised during sampling days.

#### Vegetation assessments (conditioning and responding phase)

During the second half of May 2017 (end of the conditioning phase), and again in August 2018 (responding phase), vegetation assessments were performed. In each subplot, the percentage of vegetation cover was estimated visually for each plant species within a 50 × 100 cm frame. In addition, the percentage moss cover and bare ground were recorded. The frame was placed approximately in the middle of the subplot in order to exclude potential edge effects. The cover of the different functional types (i.e. grasses, forbs) was calculated as the cumulative cover of each species belonging to the respective functional type.

#### Soil sampling for abiotic parameters and soil microbial analysis

Soils were sampled twice, once at the end of the conditioning phase, just before the vegetation was removed (June 2017) in order to establish the conditioning effects on the soil microbial community. The second sampling was used to assess whether the legacy effects persisted over time and took place roughly three months after the establishment of the responding plant community (September 2017). During both sampling events, nine soil cores (1.3 cm diameter, 10 cm depth) were taken to characterise abiotic parameters and for molecular analysis from each experimental subplot. These nine cores were then pooled per subplot and homogenised. A 2‐mL tube was filled with a subset of homogenised soil for molecular analysis at the day of sampling and stored at −80 °C. The remaining soil was used for analysis of soil abiotic parameters.

#### Soil abiotic parameters

Description of the analysis of soil abiotic parameters can be found in the Supplementary Methods.

#### Microbial DNA extraction and amplicon sequencing

DNA was extracted from 0.75 g of soil using the Power Soil DNA extraction kit (Qiagen, Hilden, Germany) according to the manufacturer’s instructions. The primers ITS4ngs and ITS3mix targeting the ITS2 region of fungal genes (Tedersoo *et al.* 2015) and the primers 515F and 806R (Caporaso *et al.* 2012; Apprill *et al.* 2015; Parada *et al.* 2016) targeting the V4 region of the 16S rRNA gene in bacteria were used in a PCR reaction (using conditions described earlier in Hannula *et al.* 2019a). The presence of PCR product of correct size was verified using agarose gel electrophoresis and the PCR products were further purified using Agencourt AMPure XP magnetic beads (Beckman Coulter, Brea, CA, USA). Adapters and barcodes were added to samples using the Nextera XT DNA library preparation kit sets A, B, and C (Illumina, San Diego, CA, USA). The final PCR product was purified again with AMPure beads, checked using agarose gel electrophoresis and quantified using a Nanodrop spectrophotometer before equimolar pooling. Based on estimated diversity levels of fungi and bacteria in these soils, we pooled all fungal samples (192) from each time point in one Illumina Miseq PE250 run and divided the bacterial samples over two separate runs (96 samples each). With two time points analysed, this resulted in two MiSeq runs for fungi and four runs for bacteria. Extraction negatives and a mock community were used and further sequenced in each sequencing run (detailed information is presented in the Supplementary Methods). Libraries were sequenced using MiSeq PE250 technology at McGill University and Genome Quebec Innovation Center.

Bacterial sequences and fungal sequences were analysed using the Hydra pipeline (version 1.3.6) and the PIPITS pipeline (version 2.3) respectively (Gweon *et al.* 2015; De Hollander 2017). Details on the settings and filtering options used can be found in the Supplementary Methods. Fungi were assigned to potential functions using FunGuild (Nguyen *et al.* 2016) and assignment was further curated using (in‐house) databases containing assignments of local grassland fungi (Tedersoo *et al.* 2015; Hannula *et al.* 2017; Mommer *et al.* 2018). We used broad guild assignments covering 64% of the sequences (‘potential plant pathogens’, ‘AMF’ and ‘saprotrophs’) for further analysis (Figure S1). For potential plant‐pathogenic fungi, the target plant species/functional group was checked based on available literature (Watanabe 2018). The sequences created in this study are deposited to the European Nucleotide Archive under accession number PRJEB31856 (available at https://www.ebi.ac.uk/ena/data/view/PRJEB31856).

#### Multivariate analyses of soil abiotic parameters, and soil fungal, bacterial and plant communities

We tested the effects of conditioning time, conditioning plant community and forb:grass ratio including all interactions on ***soil abiotic composition*** (soil nutrients and including soil pH) with a permutational analysis of variance (permanova; 999 permutations) using Euclidean distances. Furthermore, the effects of conditioning time, conditioning plant community, and forb:grass ratio and all possible interactions on ***soil fungal*** (ITS2), ***soil bacterial*** (16S), and ***plant community composition*** were assessed (permanova; 999 permutations) using Bray‐Curtis dissimilarity. Fungal and bacterial data were transformed using Hellinger transformation and plant data was square root‐transformed and standardised using Wisconsin double standardisation prior to calculating Bray–Curtis dissimilarities. Plot number was included in the models as a random effect to indicate that the one‐ and two‐year conditioned subplots belong to the same plot. All multivariate analyses were performed in R (version 3.5.3), using the ‘*vegan*’ package (version 2.5.6; R Core Team 2018; Oksanen *et al.* 2018) and community composition was visualised using ordination based on non‐metric multidimensional scaling, using the ‘*ggplot2*’ package (version 3.1.0; Wickham 2016).

To assess whether responding plant communities responded to conditioning time and forb:grass ratio, and whether particular responding plant species drove these responses, we performed (restricted) redundancy analyses with either forb:grass ratio (categorical), or conditioning time (categorical) as explanatory variables. These analyses were performed and visualised for each of the six conditioning communities separately. Redundancy analyses and visualisations were performed in Canoco 5.03 (Microcomputer Power, Ithaca NY, USA).

#### Conditioning effects on soil fungal guilds and on plant cover

We tested the effects of conditioning time, conditioning plant community, and forb:grass ratio including all interactions on (1) relative abundances of specific groups of soil fungi (total pathogens, forb pathogens, grass pathogens, and the dominating grass pathogen *Slopeiomyces cylindrosporus* (Hornby, Slope, Gutter & Sivan; Klaubauf, Lebrun & Kraus), AMF and saprotrophs; (2) plant cover (i.e. total plant cover, forb and grass cover) with general linear mixed models. Plot number was included in the model as random effect, to indicate that one‐ and two‐year conditioned subplots belonged to the same plot. Normality and homogeneity of the residuals were checked and data were transformed when necessary (indicated in the respective summary tables). All mixed models were performed in R using the ‘*nlme*’ package (version 3.1; Pinheiro *et al.* 2018).

#### Path analysis of relationships between conditioning and responding plant communities mediated via soil abiotic and biotic parameters

We calculated Bray–Curtis dissimilarities between all samples (not restricting the analysis to treatments) for plants, fungi and bacteria with the respective transformations described above, and did the same using Euclidean distance for the abiotic parameters. All calculations were done using the ‘*vegan*’ package (R Core Team 2018; Oksanen *et al.* 2018). For plant communities, all plant species present in less than three subplots were removed prior to analysis in order to diminish the effect of rare plant species. Dissimilarity matrices during conditioning and responding phases were calculated separately. Mantel tests were carried out to explore the correlations between the distance matrices using Pearson’s correlation coefficients with 999 permutations. We further corrected the p‐values obtained from the Mantel test using a Monte‐Carlo permutation test. We tested in the path model the *a priori* assumptions that conditioning by plants will change soil fungal and bacterial communities (e.g. Morrien *et al.,* 2017; Heinen *et al.,* 2018) and abiotic conditions (e.g. Bezemer *et al.,* 2006; Zhang, Van der Putten & Veen, 2016), and that these changed soil communities and conditions in turn affect the performance of responding plant communities (plant–soil feedback, Ehrenfeld *et al.,* 2005; Van der Putten *et al.,* 2013).

#### Relationships between conditioning and responding plant species

To explore relationships between the abundance of plant species during the conditioning phase (May 2017) and the abundance of the same and other plant species in the responding phase (August 2018), we constructed correlation plots using Pearson linear correlation coefficients separately for each of the six conditioning plant communities. In these correlation plots, relationships between conditioning and responding plant species are indicative of soil‐mediated effects between the species (i.e. positive or negative plant–soil feedbacks). We included only those species that comprised greater than 0.25% average cover and that were present in at least three subplots. Furthermore, we included grass and forb cover and total plant cover of the responding plant community in the correlation plots, to reveal whether observed vegetation patterns are driven by individual conditioning or responding species. All correlation plots were corrected for multiple comparisons using a Bonferroni correction. For visualisation, only pairwise Pearson correlations with significance of *P* < 0.01 are shown. All correlation matrices were constructed using the package ‘*corrplot*’ in R (Wei & Simko 2017).

## Results

### Conditioning treatment effects, via soil, on responding plant communities

**Figure 1:**
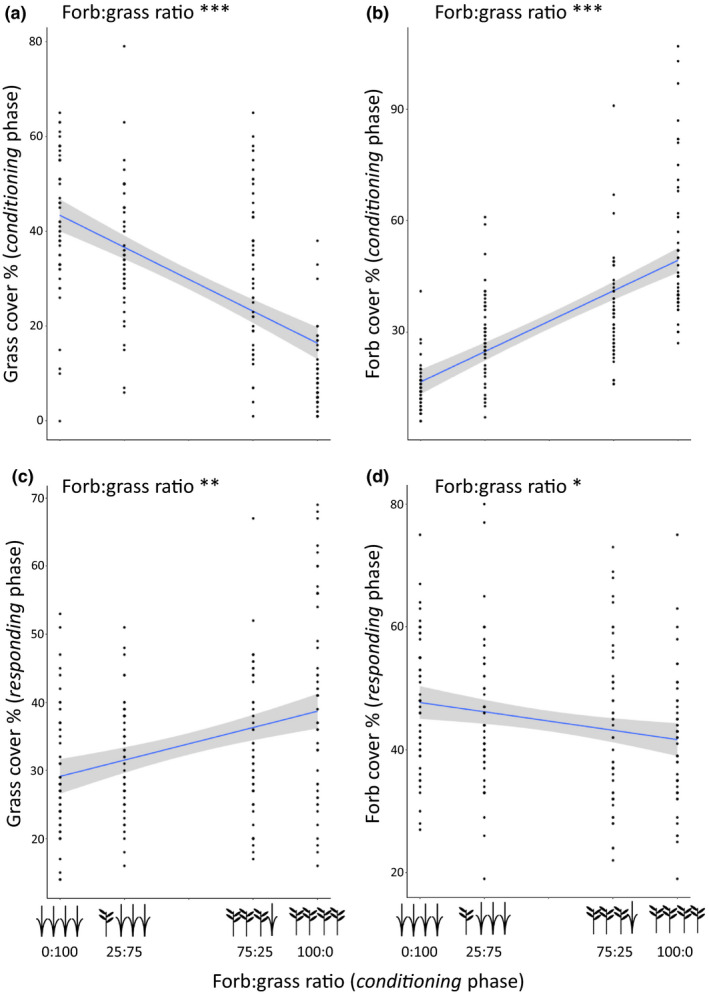
Conditioning plant communities with different forb:grass ratios create different soil legacies. Experimental manipulation of the forb:grass ratio in the conditioning phase resulted in different levels of (a) grass and (b) forb cover in the conditioning plant communities. This, in turn, created soil legacy effects that negatively affected the cover of (c) grasses and (d) forbs in the responding communities respectively. Dots represent actual data points, and a linear trend line was fitted (with a 95% confidence interval). Significant effects are presented in the figures. Asterisks represent significance levels (*P < 0.05; **P < 0.01; ***P < 0.001). Summary statistics are presented in Table S3.

Soil legacies that were created by *conditioning* treatments influenced *responding* plant communities. The forb:grass ratio of the seed mixture sown in the *conditioning* phase resulted in grass and forb covers that differed significantly from each other (Figure 1a and b). This, in turn influenced the relative abundance of grasses and forbs in the *responding* plant communities. Specifically, grass abundance in the *responding* communities was lower in plots with a legacy of higher grass abundance (Figure 1c), while forb abundance in the *responding* communities was significantly lower in plots with a legacy of higher forb abundance (Figure 1d). The pattern did not depend on plant community identity, did not differ between the two species pools, and was observed in each of the six experimental plant communities (Figures S2 and S3). Furthermore, the relationships between the relative grass and forb cover per subplot in the *conditioning* and in the *responding* phase, showed the same significant patterns (Figure S4). Conditioning time (i.e. 1 or 2 years) affected the total cover of the *responding* plant communities, with higher total cover in the plots during the responding phase after a two‐year conditioning legacy (mean cover *c*. 80% vs. *c*. 90%, Table S3).

There were significant main effects of *conditioning* plant community, forb:grass ratio and conditioning time on the *responding* plant community structure (Table S4). The effects of forb:grass ratio strongly differed between the six different conditioning plant communities, indicated by a significant interaction between the two (Table S4, Figure S5). The forb:grass ratio significantly affected responding plant community structure in three out of six conditioning communities. In the affected communities, *responding* species of a respective functional type were often negatively associated with the respective abundance of that functional type in the *conditioning* phase (Figure S6).

Conspecific (i.e. when the conditioning and responding species are the same) and heterospecific (i.e. when the conditioning species differ from the responding species) soil legacy effects were assessed and visualised using correlation plots including *conditioning* and *responding* plant species (Figure S7). There were only a limited number of (predominantly positive) conspecific effects, and these effects were not consistent between the six plant communities. For instance, we observed positive conspecific relationships for *Rumex acetosella* L. (in community 4), *Clinopodium vulgare* L. (in community 5), *Taraxacum officinale* and *Holcus lanatus* (both in community 6). Only one negative conspecific relationship was observed, for *Anthoxanthum odoratum* L. (in community 5). Furthermore, there were heterospecific relationships between *conditioning* plant species and other *responding* plant species in each of the experimental communities (Figure S7). Finally, there were *conditioning* plant species that had a strong effect on grass or forb cover in the *responding* phase. For instance, in community 2, cover of *A. millefolium* in the *conditioning* plant community positively – and *H. lanatus* negatively – affected grass cover in the *responding* plant communities. In community 3, *A. millefolium* and *Briza media* L. cover in the *conditioning* plant community negatively affected forb cover in the responding communities. In community 5, *Festuca ovina* L. cover in the *conditioning* plant community negatively affected total grass cover in the *responding* community (Figure S7).

### Conditioning treatment effects on soil communities and abiotic parameters

**Figure 2:**
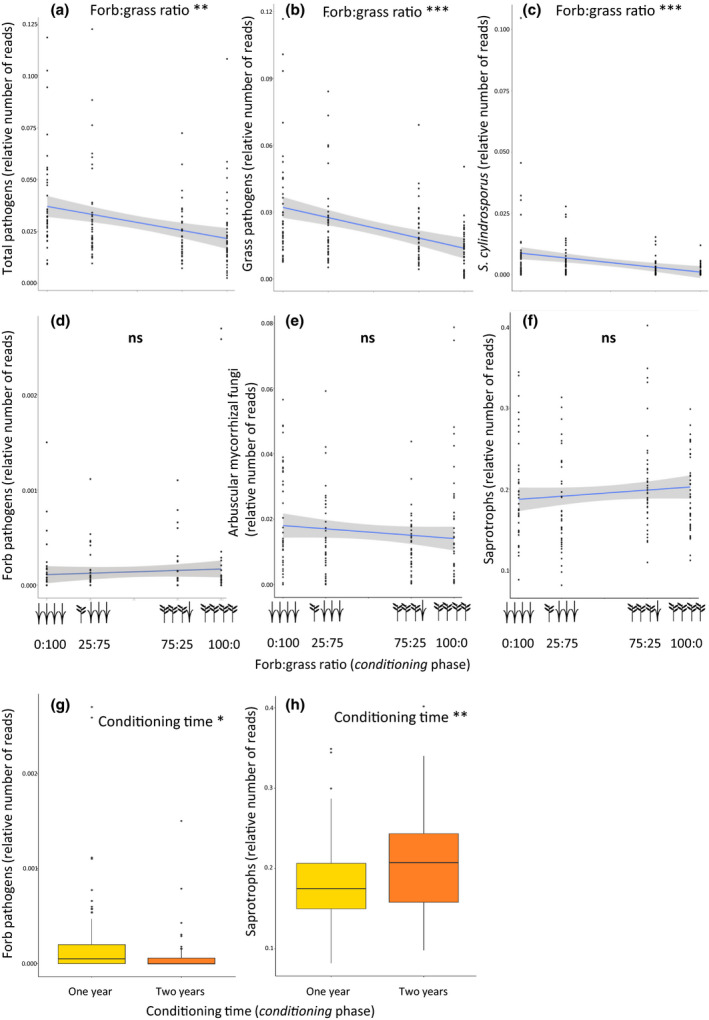
Conditioning plant communities accumulate functionally different fungal communities. Different fungal guilds were affected by different experimental treatments. The forb:grass ratio in the conditioning plant communities altered the accumulation of (a) fungal pathogens, which were predominantly (b) grass‐associated fungal pathogens, and which were rich in (c) the grass‐associated fungal pathogen *Slopeiomyces cylindrosporus*. The forb:grass ratio in the conditioning plant communities did not affect (d) forb‐associated fungal pathogens, (e) arbuscular mycorrhizal fungi, or (f) saprotrophic fungi. Conditioning time only affected the levels of (g) forb‐associated fungal pathogens, and (h) saprotrophic fungi. Dots represent actual data points, and a linear trendline was fitted (with a 95% confidence interval). Significant effects are presented in the figures. Asterisks represent significance levels (*P < 0.05; **P < 0.01; ***P < 0.001). Summary statistics are presented in Table S5.

When the cover of grasses was experimentally increased, the relative abundance of soil pathogenic fungi increased concomitantly (Figure 2a, Figure S8, Table S5). Grass pathogens dominated the total pathogens and were in turn dominated by *S. cylindrosporus*, showed an increase in relative abundance with an increase in grass cover (Figures 2b,c, Figure S8, Table S5). Forb‐specific pathogens, arbuscular mycorrhizal fungi and saprotrophs were not affected by the experimental manipulation of forb:grass ratio (Figure 2d,e and f, Figure S8). However, forb pathogens had a significantly higher relative abundance in one than in two‐year legacies (Figure 2g, Table S5), while the relative abundance of saprotrophs was higher in plots with two‐year legacy than in plots with one‐year legacy (Figure 2h, Table S5). Arbuscular mycorrhizal fungi were not affected by conditioning time (Table S5).

After the *conditioning* phase, the soil bacterial community structure was significantly affected by conditioning plant community identity and conditioning time (Table S6, Figure S9). The soil fungal community structure was significantly affected by conditioning plant community identity, conditioning time and by forb‐grass ratio, and the effect of forb‐grass ratio differed between different conditioning plant communities with different identities of plants (Table S6, Figure S10). The structure of soil abiotic parameters was affected by conditioning community identity and conditioning time (Table S6).

### Soil‐mediated pathways between conditioning and responding plant communities

**Figure 3:**
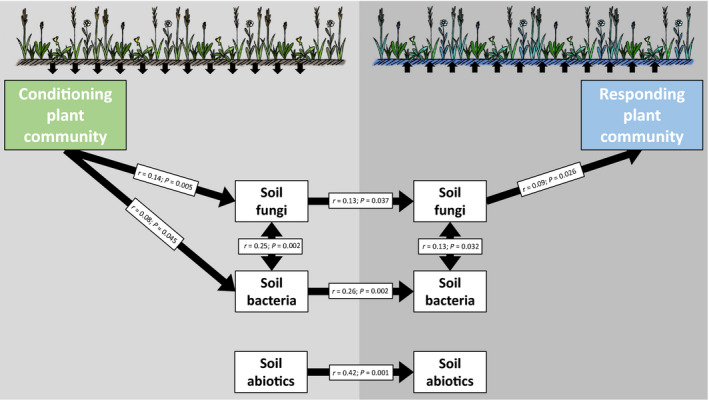
Soil legacy effects of conditioning plant communities on responding plant communities are mediated by soil fungi. A path analysis shows the relationships between conditioning plant communities and responding plant communities in the plant–soil feedback field experiment, via soil fungal and bacterial communities and soil abiotic parameters. All subplots are included in the analysis. Arrows represent significant correlations (Mantel *r* and *P*‐values) between Bray–Curtis dissimilarity matrices of plant and soil microbial communities and Euclidean distances for soil abiotic parameters.

The composition of the *conditioning* plant communities significantly explained the composition of the *responding* plant communities (Mantel test, *r* = 0.18, *P* < 0.001). The fact that these two plant communities were separated in time indicates that the effects of the *conditioning* plant communities on the responding plant communities must be mediated via the soil legacies. We used a path analysis based on Mantel tests on dissimilarity matrices to explore which components of the soil are affected by the *conditioning* plant communities and which components explain the *responding* plant communities. The composition of the *conditioning* plant community significantly explained the community composition of soil fungi and bacteria in the *conditioning* phase, but did not explain the composition of soil abiotic parameters (Figure 3). Importantly, microbial and soil abiotic parameters measured at the end of the *conditioning* phase significantly explained these parameters measured again three months after the *responding* phase had started. The composition of the soil fungal community but not that of bacteria or abiotic parameters measured in the *responding* phase correlated with the composition of the *responding* plant communities (Figure 3).

## Discussion

Here, we show in a field experiment that compositionally different plant communities create legacies in the soil that, in turn, alter the composition of subsequent plant communities that establish in these soils. Plant communities with different ratios of grasses and forbs created unique soil microbiomes, and these effects were most notable in the soil fungal community. These fungal soil legacies, in turn, affected the responding plant communities. Specifically, both grass and forb abundances in the responding phase were negatively affected by their respective abundance in the previous plant community and this effect was mediated by soil processes. We show that manipulating the composition of the vegetation in grasslands alters the microbiome in the soil, and that this alters the succeeding vegetation.

Plant communities dominated by species of a certain functional type create legacies that negatively impact plants from the same functional type. This result is very robust, as the same pattern was observed in all six plant communities that were used to condition the soil in this field experiment. This finding is also in strong agreement with previous work from artificial/pot studies (Kulmatiski *et al.* 2008; Petermann *et al.* 2008; De Kroon *et al.* 2012; Wubs & Bezemer, 2018). The functional type of a plant also has a strong effect on the community structure of soil fungi (Kos *et al.* 2015; Heinen *et al.* 2018; Hannula *et al.* 2019b). We hypothesised that manipulation of the composition of the conditioning plant communities would result in different microbial soil legacies mainly due to accumulation of specialised soil pathogens and mutualists such as arbuscular mycorrhizal fungi. We detected that, despite their overall low relative abundance at least in our study, fungal plant pathogens in the soil seem to play an important role in modulating the composition of plant communities. Contrary to our hypothesis, we did not detect a consistent contribution of AMF in these soil legacies and in their role in influencing plant communities. Earlier findings show that the composition of the AMF community in the soil highly depend on the composition of the plant species that grow in the soil and not on the functional groups these plants belong to, and that effects on and of AMF may be masked in multi‐species plant communities (Morrien *et al.* 2017; Mommer *et al.* 2018). Moreover the sampling of soils, not roots, may have played a role, as AMF are less easily detectable in soils than in roots (Saks *et al* *.* 2014).

In the soils of plant communities that had more grasses, we found an accumulation of fungal pathogens (dominated by grass‐associated fungal pathogens). Interestingly, the relative abundance of forb‐associated pathogens was very low and there was no relationship with the abundance of forbs in the vegetation. Forbs are a broad phylogenetic group (comprised of many plant families). Forb pathogens that specialise on a specific family or group of forb species are unlikely to accept hosts from all forb families, and as a result the relative abundances of such specific forb pathogens may not drive the abundance of this functional group as a whole. Grasses, on the other hand, are phylogenetically more closely related to each other (all Poaceae). Due to this higher relatedness, pathogens specialised in this group are more likely to affect a larger proportion of the functional group as a whole. While some pathogens have a rather broad host range, even specialised pathogens may attack a range of host plants if they are closely related (Barrett & Heil 2012). This may explain why accumulation of grass‐associated pathogens negatively affected grass abundance in the field, while no general pattern was detected for forbs. Importantly, our results indicate that negative soil legacy effects on grasses observed in mid‐successional grasslands, can be, at least partially, explained by accumulation of pathogens (Kulmatiski *et al.* 2008; Van der Putten *et al.* 2013).

Our results further reveal that both bacteria and fungi in the soil respond to the conditioning plant communities that grow in the soil. The effects on fungal communities, but not on the bacterial communities or abiotic characteristics of the soil, are longer‐lasting, and have knock‐on effects on the subsequent responding plant communities (Kardol *et al.* 2006). We may conclude that soil bacterial communities, although responsive to conditioning treatments, play a less important role in affecting the community dynamics of responding plant communities. As the soil communities were sampled in September 2017, three months after the conditioning vegetation was removed, the original conditioning effects on soil bacteria may have disappeared. This is in strong agreement with recent findings that soil fungal communities are shaped over time by plants, whereas bacterial communities are shaped far less strongly by plants, and instead more by varying environmental conditions over time (Hannula *et al.* 2019b). Soil legacy effects in natural plant communities are likely not driven by one taxon specifically, but rather by the composition of the soil fungal community as a whole (Semchenko *et al.* 2018; Bennett & Klironomos 2018; Mommer *et al.* 2018, but see Harrison & Bardgett, 2010). Importantly, we show that conditioning effects of plant communities on soil biota, outweigh the effects on soil abiotic parameters, and are drivers of soil legacy effects on plant growth in the field.

One potential confounding factor in the results is that plant roots and seeds originating from the conditioning plant community could have been left behind in the soil after the conditioning community was removed and that these roots may have influenced the composition of the responding communities, either directly via regrowth or via affecting the soil. There were some positive conspecific relationships between *conditioning* and *responding* plant species, but these effects were community‐specific. For instance, a positive conspecific relationship was observed for *R. acetosella*. This species flowers very quickly and produces many seeds. It is therefore plausible that seeds produced during the conditioning phase, and that entered the seedbank, caused an increased local abundance of this species in the responding communities. Furthermore, we observed a positive conspecific relationship for *C. vulgare* and *H. lanatus*. Both species regrow from root systems in pot experiments (R. Heinen, pers. obs.) and hence for these species regrowth may be responsible for these observed relationships. However, it is unlikely that these effects have had a strong effect on the *responding* plant community as a whole, as the strongest relationships – observed between functional types in the *conditioning* versus the *responding* plant communities – were negative and thus cannot be explained by regrowth or seed production. We therefore conclude that soil legacy effects must be the dominant driver of these effects.

It is important to note that at the plant species level, we detected very few indicators for conspecific plant–soil feedbacks. This is an interesting finding as the field site used in this study has been used to collect soil from for countless plant–soil feedback studies over the past decades. In the majority of these studies, plant species grown in soils from this site have negative conspecific feedback effects (e.g. Wubs & Bezemer, 2016; Heinen *et al.* 2018). This indicates that individual plant–soil feedbacks as observed in pot studies, may be counter balanced by other plant species that simultaneously grow in (and thus condition) the soil in natural and diverse plant communities. We speculate that conspecific plant–soil feedbacks could play a larger role in less diverse or more disturbed systems such as dune vegetation. However, future work is needed to investigate the role of plant diversity in plant–soil feedbacks in the field.

In conclusion, we show that the ratios between plants of different functional types within a plant community mediate plant‐induced microbial soil legacies, and that these legacies determine the composition of later establishing plant communities in the field. Importantly, this means that by managing current plant communities in the field, we can influence the composition of future plant communities and the ecological functions they provide. This opens new avenues for optimising nature management practices, which is vitally important in the face of global change, for instance in making nature more robust to climate change or invasions.

## AUTHOR CONTRIBUTIONS

TMB designed the field experiment. RH, FZ, MH and TMB, executed the first phase of the field experiment. All authors contributed to maintenance of the second phase of the experiment. RH, SEH, JDL, FZ, KS, MH, RJ and TMB collected field data. SEH and RH analysed data. RH led the writing of the manuscript, in close collaboration with SEH, JDL and TMB. All co‐authors contributed critically to the manuscript and approved the final version for publication.

## COMPETING INTERESTS

Authors declare no competing interests.

## DATA ACCESSIBILITY STATEMENT

Data will be made available on Dryad upon publication. Sequence data have been deposited in the European Nucleotide Archive and will be made available upon publication (https://doi.org/10.5061/dryad.70rxwdbtg).

## Supporting information

Supplementary Material

## References

[ele13497-bib-0001] Apprill, A. , McNally, S. , Parsons, R. & Weber, L. (2015). Minor revision to V4 region SSU rRNA 806R gene primer greatly increases detection of SAR11 bacterioplankton. Aquat. Microb. Ecol., 75, 129–137.

[ele13497-bib-0002] Bardgett, R.D. & Van der Putten, W.H. (2014). Belowground biodiversity and ecosystem functioning. Nature, 515, 505–511. 25428498 10.1038/nature13855

[ele13497-bib-0003] Barrett, L.G. & Heil, M. (2012). Unifying concepts and mechanisms in the specificity of plant–enemy interactions. Trends Plant Sci., 17, 282–292. 22465042 10.1016/j.tplants.2012.02.009

[ele13497-bib-0004] Bennett, J.A. & Klironomos, J. (2018). Mechanisms of plant–soil feedback: interactions among biotic and abiotic drivers. New Phytol., 222, 91–96. 30451287 10.1111/nph.15603

[ele13497-bib-0005] Bezemer, T.M. , Lawson, C.S. , Hedlund, K. , Edwards, A.R. , Brook, A.J. , Igual, J. M. *et al* (2006). Plant species and functional group effects on abiotic and microbial soil properties and plant–soil feedback responses in two grasslands. J Ecol., 94, 893–904.

[ele13497-bib-0006] Caporaso, J.G. , Lauber, C.L. , Walters, W.A. , Berg‐Lyons, D. , Huntley, J. , Fierer, N. *et al* (2012). Ultra‐high‐throughput microbial community analysis on the Illumina HiSeq and MiSeq platforms. ISME J., 6, 1621–1624. 22402401 10.1038/ismej.2012.8 PMC3400413

[ele13497-bib-0007] Casper, B.B. & Castelli, J.P. (2007). Evaluating plant–soil feedback together with competition in a serpentine grassland. Ecol. Lett., 10, 394–400. 17498138 10.1111/j.1461-0248.2007.01030.x

[ele13497-bib-0008] Craine, J. , Froehle, J. , Tilman, D. , Wedin, D. & Chapin, F.S. III (2001). The relationships among root and leaf traits of 76 grassland species and relative abundance along fertility and disturbance gradients. Oikos, 93, 274–285.

[ele13497-bib-0009] Craine, J.M. , Tilman, D. , Wedin, D. , Reich, P. , Tjoelker, M. & Knops, J. (2002). Functional traits, productivity and effects on nitrogen cycling of 33 grassland species. Funct. Ecol., 16, 563–574.

[ele13497-bib-0010] De Kroon, H. , Hendriks, M. , Van Ruijven, J. , Ravenek, J. , Padilla, F.M. , Jongejans, E. *et al* (2012). Root responses to nutrients and soil biota: drivers of species coexistence and ecosystem productivity. J. Ecol., 100, 6–15.

[ele13497-bib-0011] De Long, J.R. , Heinen, R. , Steinauer, K. , Hannula, S.E. , Huberty, M. , Jongen, R. *et al* (2019). Taking plant–soil feedbacks to the field in a temperate grassland. Basic Appl. Ecol., 40, 30–42.

[ele13497-bib-0012] De Hollander, M. , nioo‐knaw, hydra: 1.3.3 . (2017). doi: 10.5281/zenodo.884028

[ele13497-bib-0013] Diez, J.M. , Dickie, I. , Edwards, G. , Hulme, P.E. , Sullivan, J.J. & Duncan, R.P. (2010). Negative soil feedbacks accumulate over time for non‐native plant species. Ecol. Lett., 13, 803–809. 20482584 10.1111/j.1461-0248.2010.01474.x

[ele13497-bib-0014] Ehrenfeld, J.G. , Ravit, B. & Elgersma, K.J. (2005). Feedback in the plant‐soil system. Annu. Rev. Environ. Resour., 30, 75–115.

[ele13497-bib-0015] Eppinga, M.B. , Baudena, M. , Johnson, D.J. , Jiang, J. , Mack, K.M.L. , Strand, A.E. *et al* (2018). Frequency‐dependent feedback constrains plant community coexistence. Nat. Ecol. Evol., 2, 1403–1407. 30061563 10.1038/s41559-018-0622-3

[ele13497-bib-0016] Gweon, H.S. , Oliver, A. , Taylor, J. , Booth, T. , Gibbs, M. , Read, D.S. *et al* (2015). PIPITS: an automated pipeline for analyses of fungal internal transcribed spacer sequences from the Illumina sequencing platform. Methods. Ecol. Evol., 6, 973–980. 27570615 10.1111/2041-210X.12399 PMC4981123

[ele13497-bib-0017] Hannula, S.E. , Morrien, E. , De Hollander, M. , Van der Putten, W.H. , Van Veen, J.A. & De Boer, W. (2017). Shifts in rhizosphere fungal community during secondary succession following abandonment from agriculture. ISME J, 11, 2294–2304. 28585935 10.1038/ismej.2017.90 PMC5607372

[ele13497-bib-0018] Hannula, S.E. , Zhu, F. , Heinen, R. & Bezemer, T.M. (2019a). Foliar‐feeding insects acquire microbiomes from the soil rather than the host plant. Nat. Commun., 10, 1254. 30890706 10.1038/s41467-019-09284-w PMC6425034

[ele13497-bib-0019] Hannula, S.E. , Kielak, A.M. , Steinauer, K. , Huberty, K. , Jongen, R. , De Long, J.R. *et al* (2019b). Time after time: temporal variation in the effects of grass and forb species on soil bacterial and fungal communities. MBio, 10, e02635–19. 31848279 10.1128/mBio.02635-19 PMC6918080

[ele13497-bib-0020] Harrison, K.A. & Bardgett, R.D. (2010). Influence of plant species and soil conditions on plant–soil feedback in mixed grassland communities. J. Ecol., 98, 384–395.

[ele13497-bib-0021] Heinen, R. , van der Sluijs, M. , Biere, A. , Harvey, J.A. & Bezemer, T.M. (2018). Plant community composition but not plant traits determine the outcome of soil legacy effects on plants and insects. J. Ecol., 106, 1217–1229.

[ele13497-bib-0022] Heinen, R. , Biere, A. & Bezemer, T.M. (2019). Plant traits shape soil legacy effects on individual plant–insect interactions. Oikos, 129, 261–273.

[ele13497-bib-0023] Jeffery, S. , Memelink, I. , Hodgson, E. , Jones, S. , Van de Voorde, T.F.J. , Bezemer, T.M. *et al* (2017). Initial biochar effects on plant productivity derive from N fertilization. Plant Soil, 415, 435–448.

[ele13497-bib-0024] Kardol, P. , Bezemer, T.M. & Van Der Putten, W.H. (2006). Temporal variation in plant–soil feedback controls succession. Ecol. Lett., 9, 1080–1088. 16925657 10.1111/j.1461-0248.2006.00953.x

[ele13497-bib-0025] Kardol, P. , Cornips, N.J. , van Kempen, M.M. , Bakx‐Schotman, J.T. & Van der Putten, W.H. (2007). Microbe‐mediated plant–soil feedback causes historical contingency effects in plant community assembly. Ecol. Monogr., 77, 147–162.

[ele13497-bib-0026] Kos, M. , Tuijl, M.A. , de Roo, J. , Mulder, P.P. & Bezemer, T.M. (2015). Species‐specific plant–soil feedback effects on above‐ground plant–insect interactions. J. Ecol., 103, 904–914.

[ele13497-bib-0027] Kulmatiski, A. , Beard, K.H. , Stevens, J.R. & Cobbold, S.M. (2008). Plant–soil feedbacks: a meta‐analytical review. Ecol. Lett., 11, 980–992. 18522641 10.1111/j.1461-0248.2008.01209.x

[ele13497-bib-0028] Latz, E. , Eisenhauer, N. , Scheu, S. & Jousset, A. (2015). Plant identity drives the expression of biocontrol factors in a rhizosphere bacterium across a plant diversity gradient. Funct. Ecol., 29, 1225–1234.

[ele13497-bib-0029] Latz, E. , Eisenhauer, N. , Rall, B.C. , Scheu, S. & Jousset, A. (2016). Unravelling linkages between plant community composition and the pathogen‐suppressive potential of soils. Sci. Rep., 6, 23584. 27021053 10.1038/srep23584 PMC4810420

[ele13497-bib-0030] Lekberg, Y. , Bever, J.D. , Bunn, R.A. , Callaway, R.M. , Hart, M.M. , Kivlin, S.N. *et al* (2018). Relative importance of competition and plant–soil feedback, their synergy, context dependency and implications for coexistence. Ecol. Lett., 21, 1268–1281. 29896848 10.1111/ele.13093

[ele13497-bib-0031] Mommer, L. , Cotton, T.A. , Raaijmakers, J.M. , Termorshuizen, A.J. , Van Ruijven, J. , Hendriks, M. *et al* (2018). Lost in diversity: the interactions between soil‐borne fungi, biodiversity and plant productivity. New Phytol., 218, 542–553. 29468690 10.1111/nph.15036 PMC5887887

[ele13497-bib-0032] Morriën, E. , Hannula, S.E. , Snoek, L.B. , Helmsing, N.R. , Zweers, H. *et al* (2017). Soil networks become more connected and take up more carbon as nature restoration progresses. Nat. Commun., 8, 14349. 28176768 10.1038/ncomms14349 PMC5309817

[ele13497-bib-0033] Nguyen, N.H. , Song, Z. , Bates, S.T. , Branco, S. , Tedersoo, L. , Menke, J. *et al* (2016). FUNGuild: An open annotation tool for parsing fungal community datasets by ecological guild. Fungal Ecol., 20, 241–248.

[ele13497-bib-0034] Oksanen, J. , Blanchet, F.G. , Kindt, R. , Legendre, P. , O’Hara, R.B. , Simpson, G.L. *et al* (2018). Vegan: Community Ecology Package, https://CRAN.R‐project.org/package=vegan.

[ele13497-bib-0035] Parada, A.E. , Needham, D.M. & Fuhrman, J.A. (2016). Every base matters: assessing small subunit rRNA primers for marine microbiomes with mock communities, time series and global field samples. Environ. Microbiol., 18, 1403–1414. 26271760 10.1111/1462-2920.13023

[ele13497-bib-0036] Petermann, J.S. , Fergus, A.J. , Turnbull, L.A. & Schmid, B. (2008). Janzen‐Connell effects are widespread and strong enough to maintain diversity in grasslands. J. Ecol., 89, 2399–2406. 10.1890/07-2056.1 18831160

[ele13497-bib-0037] Philippot, L. , Raaijmakers, J.M. , Lemanceau, P. & Van der Putten, W.H. (2013). Going back to the roots: the microbial ecology of the rhizosphere. Nat. Rev. Microbiol., 11, 789–799. 24056930 10.1038/nrmicro3109

[ele13497-bib-0038] Pinheiro, J. , Bates, D. , DebRoy, S. & Sarkar, D. , R Core Team (2018). Nlme: Linear and Nonlinear Mixed Effects Models. https://CRAN.R‐project.org/package=nlme.

[ele13497-bib-0039] R Core Team (2018). R: A Language and Environment for Statistical Computing. R Foundation for Statistical Computing, Vienna, Austria https://www.R‐project.org/.

[ele13497-bib-0040] Ravenek, J.M. , Mommer, L. , Visser, E.J. , van Ruijven, J. , van der Paauw, J.W. , Smit‐Tiekstra, A. *et al* (2016). Linking root traits and competitive success in grassland species. Plant Soil, 407, 39–53.

[ele13497-bib-0041] Reynolds, H.L. , Packer, A. , Bever, J.D. & Clay, K. (2003). Grassroots ecology: plant–microbe–soil interactions as drivers of plant community structure and dynamics. Ecology, 84, 2281–2291.

[ele13497-bib-0042] Rooij‐van, De , der Goes, P.C.E.M. , Peters, B.A.M. & Van der Putten, W.H. (1998). Vertical migration of nematodes and soil‐borne fungi to developing roots of *Ammophila arenaria* (L.) link after sand accretion. Appl. Soil Ecol., 10, 1–10.

[ele13497-bib-0043] Saks, Ü. , Davison, J. , Öpik, M. , Vasar, M. , Moora, M. & Zobel, M. (2014). Root‐colonizing and soil‐borne communities of arbuscular mycorrhizal fungi in a temperate forest understorey. Botany, 92, 277–285.

[ele13497-bib-0044] Semchenko, M. , Leff, J.W. , Lozano, Y.M. , Saar, S. , Davison, J. , Wilkinson, A. *et al* (2018). Fungal diversity regulates plant‐soil feedbacks in temperate grassland. Sci. Adv., 4, eaau4578. 30498781 10.1126/sciadv.aau4578 PMC6261650

[ele13497-bib-0045] Semchenko, M. , Nettan, S. , Sepp, A. , Zhang, Q. , Abakumova, M. , Davison, J. *et al* (2019). Soil biota and chemical interactions promote co‐existence in co‐evolved grassland communities. J. Ecol., 107, 2611–2622.

[ele13497-bib-0046] Tedersoo, L. , Anslan, S. , Bahram, M. , Põlme, S. , Riit, T. , Liiv, I. *et al* (2015). Shotgun metagenomes and multiple primer pair‐barcode combinations of amplicons reveal biases in metabarcoding analyses of fungi. MycoKeys, 10, 1–43.

[ele13497-bib-0047] Teste, F.P. , Kardol, P. , Turner, B.L. , Wardle, D.A. , Zemunik, G. , Renton, M. *et al* (2017). Plant‐soil feedback and the maintenance of diversity in Mediterranean‐climate shrublands. Science, 355, 173–176. 28082588 10.1126/science.aai8291

[ele13497-bib-0048] Tjoelker, M. , Craine, J.M. , Wedin, D. , Reich, P.B. & Tilman, D. (2005). Linking leaf and root trait syndromes among 39 grassland and savannah species. New Phytol., 167, 493–508. 15998401 10.1111/j.1469-8137.2005.01428.x

[ele13497-bib-0049] Van de Voorde, T.F. , Van der Putten, W.H. & Bezemer, T.M. (2011). Intra‐and interspecific plant–soil interactions, soil legacies and priority effects during old‐field succession. J. Ecol., 99, 945–953.

[ele13497-bib-0050] Van der Putten, W.H. , Bardgett, R.D. , Bever, J.D. , Bezemer, T.M. , Casper, B.B. , Fukami, T. *et al* (2013). Plant–soil feedbacks: the past, the present and future challenges. J. Ecol., 101, 265–276.

[ele13497-bib-0051] Watanabe, T. (2018). Pictorial atlas of soilborne fungal plant pathogens and diseases. CRC Press, Taylor and Francis Group. https://www.crcpress.com/Pictorial‐Atlas‐of‐Soilborne‐Fungal‐Plant‐Pathogens‐and‐Diseases/Watanabe/p/book/9781138294592.

[ele13497-bib-0052] Wei, T. & Simko, V. (2017). R package "corrplot": Visualization of a correlation matrix. https://CRAN.R‐project.org/package=corrplot.

[ele13497-bib-0053] Wickham, H. (2016). Ggplot2: Elegant Graphics for Data Analysis. Springer‐Verlag, New York, NY https://CRAN.R‐project.org/package=ggplot2.

[ele13497-bib-0054] Wubs, E.R.J. & Bezemer, T.M. (2016). Effects of spatial plant–soil feedback heterogeneity on plant performance in monocultures. J. Ecol., 104, 364–376.

[ele13497-bib-0055] Wubs, E.R.J. & Bezemer, T.M. (2018). Plant community evenness responds to spatial plant–soil feedback heterogeneity primarily through the diversity of soil conditioning. Funct. Ecol., 32, 509–521.

[ele13497-bib-0056] Wubs, E.R.J. , Van der Putten, W.H. , Bosch, M. & Bezemer, T.M. (2016). Soil inoculation steers restoration of terrestrial ecosystems. Nat. Plants, 2, 1–5. 10.1038/nplants.2016.107 27398907

[ele13497-bib-0057] Wubs, E.R.J. , Van der Putten, W.H. , Mortimer, S.R. , Korthals, G.W. , Duyts, H. , Wagenaar, R. *et al* (2019). Single introductions of soil biota and plants generate long‐term legacies in soil and plant community assembly. Ecol. Lett., 22, 1145–1151. 31020756 10.1111/ele.13271 PMC6850328

[ele13497-bib-0058] Zhang, N. , Van der Putten, W.H. & Veen, G.F. (2016). Effects of root decomposition on plant–soil feedback of early‐and mid‐successional plant species. New Phytol., 212, 220–231. 27214646 10.1111/nph.14007

