# Supplementary material for: Plant community composition steers grassland vegetation via soil legacy effects
Source: Ecol Lett. 2020 Apr 7;23(6):973–82. doi: 10.1111/ele.13497 (PMC7318629; doi:10.1111/ele.13497)

Supplementary Information to:

**Plant community composition steers grassland vegetation via soil legacy effects**

Robin Heinen^1,2,3#^, S. Emilia Hannula^1,#^, Jonathan R. De Long^1,#^, Martine Huberty^1,2^, Renske Jongen^1^, Anna Kielak^1^, Katja Steinauer^1^, Feng Zhu^1,4^ and T. Martijn Bezemer^1,2,#^

^1^ Department of Terrestrial Ecology, Netherlands Institute of Ecology, P.O. Box 50, 6700 AB, Wageningen, The Netherlands

^2^ Institute of Biology, Section Plant Ecology and Phytochemistry, Leiden University, P.O. Box 9505, 2300 RA Leiden, The Netherlands

^3^ *Current address:* Lehrstuhl für Terrestrische Ökologie, Wissenschaftszentrum Weihenstephan für Ernährung, Landnutzung und Umwelt, Technische Universität München, Hans-Carl-von-Carlowitz-Platz 2, D-85354, Freising, Germany

^4^ Key Laboratory of Agricultural Water Resources, Hebei Key Laboratory of Soil Ecology, Center for Agricultural Resources Research, Institute of Genetic and Developmental Biology, The Chinese Academy of Sciences, 286 Huaizhong Road, 050021, Shijiazhuang, Hebei, China

^#^ These authors contributed equally to this manuscript

Author for correspondence: Robin Heinen ([R.Heinen@nioo.knaw.nl](mailto:R.Heinen@nioo.knaw.nl); [robin.heinen@tum.de](mailto:robin.heinen@tum.de))

**Supplementary Methods:**

*Soil abiotic parameters*

Soil samples used to measure soil abiotic characteristics were dried at 40 °C and sieved through a 2 mm sieve. Three grams of dried soil was weighed exactly and mixed with 30 ml of 0.01 M CaCl_2_. The mixture was shaken for 2 h on a mechanical shaker with linear movement at 250 rpm. The samples were centrifuged for 5 m at 3000 rpm. Then, 15 mL of the supernatant was filtered through a Whatman Puradisc Aqua 30 syringe filter with cellulose acetate membrane. Subsequently, 12.87 mL of the filtrate and 130 μL HNO_3_ were mixed in a clean 15 mL tube. The sample was mixed using a vortex and analyzed for soil extractable micronutrients (Fe, K, Mg, P, S, Zn) using an inductively coupled plasma - optical emission spectrometer (ICP-OES, Thermo Scientific iCAP 6500 Duo Instrument with axial and radial view and CID detector microwave digestion system). The remaining part of the filtrate was transferred to a 15 mL tube to measure soil pH, NO_2_+NO_3_ and NH_4_. After recording the pH (inoLab pH 7310), the soil extracts were analyzed on a QuAAtro Autoanalyzer (Seal Analytical, Mequon, Wisconsin, USA).

*Bioinformatics analysis*

Both fungal and bacterial sequences were paired using VSEARCH and quality was filtered using standard parameters of the pipelines. For fungi, the sequences were first paired and then filtered while for bacteria sequences were first filtered and then paired (Gweon *et al.* 2015; De Hollander 2017). For fungi, the fungal ITS2 region was extracted using ITSx and the analysis was done using the trained dataset of ITS regions derived from UNITE database (Bengtsson-Palme *et al.* 2013), also removing all sequences from non-fungal origin. For both bacteria and fungi, short sequences (<100bp) were removed and remaining sequences were clustered based on a 97% similarity threshold using VSEARCH. Fungal chimeric sequences were removed by comparing with the UNITE uchime database and bacterial chimeric sequences were removed using uchime with VSEARCH. The representative fungal sequences were identified using the RDP classifier against the UNITE database (version 02.02.2019; Abarenkov *et al.* 2010) and they were further clustered into phylotypes. The representative bacterial sequences were clustered using the SINA classification with the SILVA database (release 128).

*Filtering of microbial sequencing data*

The microbial sequencing data from the conditioning phase (June 2017) and three months after establishment of the responding phase (September 2017) were analyzed together. We first filtered out microbial taxa that were present in less than ten samples and that had an abundance of less than 0.01%. For ITS data, the sequences derived from other organisms than fungi were removed and for 16S data, sequences originating from mitochondria, chloroplasts and Archaea were removed. For both bacteria and fungi, samples with less than 1000 reads remaining were removed from the dataset and read numbers were normalized using total sum scaling (TSS).

*Mock communities and negative controls*

The used mock community consisted of 10 fungal species and associated bacteria, and was included to control for the potential variation between sequencing runs and to increase the accuracy of the bioinformatics analysis. After filtering, we detected 13 fungal OTUs that were identical in all mock communities sequenced. The abundances of these OTUs in the mock communities in different sequencing runs were highly correlated (Pearson correlation, R^2^=0.97, p<0.001). There were minute amounts (<5 species and <100 reads) of bacteria and fungi found in the negative control samples thus confirming that there was no (cross) contamination.

**Supplementary references:**

Abarenkov, K., Nilsson, R. H., Larsson, K.-H., Alexander, I. J., Eberhardt, U., Erland, S. *et al.* (2010). The UNITE database for molecular identification of fungi – recent updates and future perspectives. *New Phytol*. 186, 281-285.

Bengtsson‐Palme, J., Ryberg, M., Hartmann, M., Branco, S., Wang, Z., Godhe, A., *et al.* (2013). Improved software detection and extraction of ITS1 and ITS2 from ribosomal ITS sequences of fungi and other eukaryotes for analysis of environmental sequencing data. *Methods. Ecol. Evol*. 4, 914-919.

De Hollander, M. nioo-knaw/hydra: 1.3.3, (2017). https://doi.org/10.5281/zenodo.597131

Gweon, H. S., Oliver, A., Taylor, J., Booth, T., Gibbs, M., Read, D. S. *et al.* (2015). PIPITS: an automated pipeline for analyses of fungal internal transcribed spacer sequences from the Illumina sequencing platform. *Methods. Ecol. Evol*. 6, 973-980.

**Supplementary Tables:**

*Supplementary Table 1*. Table showing the 24 species used in the conditioning phase (indicated with a *) and the 33 species used in the feedback phase. Growth rates (fast/slow) of the two species pools are presented for the 24 species. Note: *Geranium molle* was not included in the feedback phase due to unavailability of seeds.

| Species | Family | Growth-form | Growth rate |
| --- | --- | --- | --- |
| *Achillea millefolium** | Asteraceae | forb | fast |
| *Agrostis capillaris** | Poaceae | grass | fast |
| *Alopecurus pratensis** | Poaceae | grass | fast |
| *Anthoxanthum odoratum** | Poaceae | grass | slow |
| *Arrhenatherum elatius** | Poaceae | grass | slow |
| *Briza media** | Poaceae | grass | slow |
| *Calluna vulgaris* | Ericaceae | dwarf shrub | - |
| *Clinopodium vulgare** | Lamiaceae | forb | slow |
| *Crepis capillaris** | Asteraceae | forb | fast |
| *Dactylis glomerata** | Poaceae | grass | fast |
| *Deschampsia flexuosa** | Poaceae | grass | slow |
| *Epilobium hirsutum** | Onagraceae | forb | fast |
| *Festuca filiformis* | Poaceae | grass | slow |
| *Festuca ovina** | Poaceae | grass | slow |
| *Galium mollugo** | Rubiaceae | forb | slow |
| *Galium verum* | Rubiaceae | forb | - |
| *Geranium molle** | Geraniaceae | forb | slow |
| *Gnaphalium sylvaticum** | Asteraceae | forb | slow |
| *Hieracium pilosella* | Asteraceae | forb | - |
| *Holcus lanatus** | Poaceae | grass | fast |
| *Lolium perenne** | Poaceae | grass | fast |
| *Lotus corniculatus* | Fabaceae | legume | - |
| *Luzula campestris* | Juncaceae | rush | - |
| *Myosotis arvensis** | Boraginaceae | forb | slow |
| *Nardus stricta* | Poaceae | grass | - |
| *Phleum pratense** | Poaceae | grass | fast |
| *Plantago lanceolata** | Plantaginaceae | forb | fast |
| *Potentilla erecta* | Rosaceae | forb | - |
| *Rumex acetosella** | Polygonaceae | forb | fast |
| *Sisymbrium officinale* | Brassicaceae | forb | - |
| *Succisa pratensis* | Caprifoliaceae | forb | - |
| *Taraxacum officinale** | Asteraceae | forb | fast |
| *Tripleurospermum maritimum** | Asteraceae | forb | slow |
| *Trisetum flavescens** | Poaceae | grass | slow |

*Supplementary Table 2.* Overview of the targeted plant species composition of the six plant communities that were used in the conditioning phase of the experiment. Plant species were randomly selected from two species pools that contained faster-growing or slower-growing plants, indicated by ‘fast’ or ‘slow’. After De Long et al. 2019.

| **Community** | **Species pool (fast/slow)** | **Grass species** | | | **Forb species** | | |
| --- | --- | --- | --- | --- | --- | --- | --- |
| Community 1 | Fast | *Alopecurus pratense* | *Dactylis glomerata* | *Holcus lanatus* | *Crepis capillaris* | *Plantago lanceolata* | *Taraxacum officinale* |
| Community 2 | Fast | *Agrostis capillaris* | *Holcus lanatus* | *Lolium perenne* | *Achillea millefolium* | *Crepis capillaris* | *Rumex*  *acetosella* |
| Community 3 | Fast | *Dactylis glomerata* | *Lolium perenne* | *Phleum pratense* | *Achillea millefolium* | *Epilobium hirsutum* | *Taraxacum officinale* |
| Community 4 | Slow | *Arrhenatherum elatius* | *Briza*  *media* | *Festuca ovina* | *Clinopodium vulgare* | *Geranium molle* | *Tripleurospermum maritimum* |
| Community 5 | Slow | *Anthoxanthum odoratum* | *Briza*  *media* | *Trisetum flavescens* | *Clinopodium vulgare* | *Gnaphalium sylvaticum* | *Myosotis*  *arvensis* |
| Community 6 | Slow | *Anthoxanthum odoratum* | *Deschampsia flexuosa* | *Trisetum flavescens* | *Geranium molle* | *Myosotis arvensis* | *Tripleurospermum maritimum* |

*Supplementary Table 3.* Summary statistics of a mixed model testing the effects of conditioning community, conditioning time, forb:grass ratio in the conditioning plant community, and their interactions on the responding plant community characteristics one year (August 2018) after initiating the responding phase. Presented are degrees of freedom, F-values and p-values. Significant effects (p<0.05) are presented in bold.

|  |  | Grass  cover | | Forb  cover | | Total  cover | |
| --- | --- | --- | --- | --- | --- | --- | --- |
|  | df1,df2 | F-value | p-value | F-value | p-value | F-value | p-value |
| Conditioning Community ( C ) | 5,84 | 2.16 | 0.066 | 2.20 | 0.062 | 1.01 | 0.417 |
| Conditioning Time ( T ) | 1,84 | 0.40 | 0.529 | **5.22** | **0.025** | **11.63** | **0.001** |
| Forb:Grass Ratio ( R ) | 1,84 | **19.25** | **<.001** | **7.14** | **0.009** | 0.53 | 0.469 |
| C x T | 5,84 | 1.09 | 0.373 | 0.71 | 0.619 | 0.80 | 0.552 |
| C x R | 5,84 | 0.47 | 0.797 | 0.30 | 0.911 | 0.49 | 0.781 |
| T x R | 1,84 | 2.88 | 0.094 | 0.21 | 0.646 | 0.27 | 0.603 |
| C x R x T | 5,84 | 0.52 | 0.761 | 1.66 | 0.154 | 2.25 | 0.056 |

*Supplementary Table 4.* Summary statistics of a Permanova testing the effects of conditioning community, conditioning time, forb:grass ratio in the conditioning plant community, and their interactions on the responding plant community structure one year (August 2018) after the transition from conditioning to responding phase. Presented are degrees of freedom, variance explained (R^2^), F-values and p-values. Significant effects (p<0.05) are presented in bold.

|  | df1,df2 | F-value | R^2^ | p-value |
| --- | --- | --- | --- | --- |
| Conditioning Community C | **5,191** | **4.75** | **0.111** | **0.001** |
| Conditioning Time T | **1,191** | **3.81** | **0.018** | **0.001** |
| Conditioning Forb:Grass Ratio R | **1,191** | **4.32** | **0.020** | **0.001** |
| C x T | 5,191 | 0.77 | 0.018 | 0.946 |
| C x R | **5,191** | **2.07** | **0.048** | **0.001** |
| T x R | 1,191 | 0.54 | 0.002 | 0.942 |
| C x T x R | 5,191 | 0.50 | 0.011 | 1.000 |

*Supplementary Table 5*. Summary statistics of a mixed model testing the effects of conditioning community, conditioning time, forb:grass ratio in the conditioning plant community, and their interactions on the relative abundance of total pathogenic fungi, grass-pathogenic fungi, the grass pathogen *Slopeiomyces cylindrosporus*, forb-pathogenic fungi, saprotrophic fungi, and arbuscular mycorrhizal fungi (AMF) present in the soil three months (September 2017) after the transition from conditioning to responding phase. Presented are degrees of freedom, F-values and p-values. Significant effects (p<0.05) are presented in bold. Data were transformed to values relative to total read number.

|  |  | Total Pathogens | | Grass Pathogens | | *S. cylindrosporus* | | Forb Pathogens | | Saprotrophs | | AMF | |
| --- | --- | --- | --- | --- | --- | --- | --- | --- | --- | --- | --- | --- | --- |
|  | df1,df2 | F-value | p-value | F-value | p-value | F-value | p-value | F-value | p-value | F-value | p-value | F-value | p-value |
| Conditioning Community ( C ) | 5,79 | 1.22 | 0.307 | 0.97 | 0.442 | 1.01 | 0.420 | 0.67 | 0.647 | 1.83 | 0.116 | 0.93 | 0.467 |
| Conditioning Time ( T ) | 1,45 | 3.22 | 0.078 | 2.34 | 0.132 | 2.53 | 0.117 | **6.75** | **0.012** | **9.15** | **0.004** | 2.30 | 0.135 |
| Forb:Grass Ratio ( R ) | 1,79 | **12.80** | **0.001** | **21.05** | **<.001** | **14.77** | **<.001** | 0.68 | 0.413 | 1.84 | 0.179 | 1.80 | 0.183 |
| C x T | 5,57 | 1.54 | 0.192 | 1.62 | 0.170 | 1.90 | 0.109 | 0.58 | 0.718 | 2.00 | 0.092 | 1.65 | 0.163 |
| C x R | 5,79 | 0.72 | 0.612 | 0.45 | 0.810 | 1.92 | 0.100 | 2.03 | 0.083 | 2.20 | 0.063 | 1.05 | 0.394 |
| T x R | 1,57 | 1.80 | 0.186 | **5.62** | **0.021** | 3.41 | 0.070 | 2.36 | 0.130 | 1.15 | 0.289 | 0.05 | 0.825 |
| C x R x T | 5,57 | 0.83 | 0.533 | 1.66 | 0.158 | 2.04 | 0.087 | 1.30 | 0.277 | 1.37 | 0.249 | 2.25 | 0.062 |

*Supplementary Table 6*: Summary statistics of a Permanova testing the effects of conditioning community, conditioning time, forb:grass ratio in the conditioning plant community, and their interactions on the soil bacterial and fungal community structure, and on the structure of soil abiotic parameters three months after initiating the responding phase (September 2017). Presented are degrees of freedom, variance explained (R^2^), F-values and p-values. Significant effects (p<0.05) are presented in bold.

|  | Soil bacterial community | | | | Soil fungal community | | | | Soil abiotic parameters | | | |
| --- | --- | --- | --- | --- | --- | --- | --- | --- | --- | --- | --- | --- |
|  | df1,df2 | F-value | R^2^ | p-value | df1,df2 | F-value | R^2^ | p-value | df1,df2 | F-value | R^2^ | p-value |
| Conditioning Community C | **5,107** | **1.42** | **0.066** | **0.003** | **5,162** | **1.54** | **0.046** | **0.006** | **5,191** | **2.38** | **0.059** | **0.010** |
| Conditioning Time T | **1,107** | **1.55** | **0.014** | **0.029** | **1,162** | **1.85** | **0.011** | **0.020** | **1,191** | **4.18** | **0.021** | **0.013** |
| Conditioning Forb:Grass Ratio R | 1,107 | 1.13 | 0.011 | 0.210 | **1,162** | **3.00** | **0.018** | **0.001** | 1,191 | 1.88 | 0.009 | 0.138 |
| C x T | 5,107 | 1.13 | 0.052 | 0.113 | 5,162 | 0.76 | 0.023 | 0.962 | 5,191 | 0.52 | 0.013 | 0.923 |
| C x R | 5,107 | 0.77 | 0.036 | 0.997 | **5,162** | **1.57** | **0.047** | **0.003** | 5,191 | 1.58 | 0.039 | 0.111 |
| T x R | 1,107 | 0.88 | 0.008 | 0.652 | 1,162 | 0.99 | 0.006 | 0.441 | 1,191 | 0.45 | 0.002 | 0.702 |
| C x T x R | 5,107 | 0.76 | 0.035 | 0.997 | 5,162 | 0.69 | 0.021 | 0.989 | 5,191 | 0.76 | 0.019 | 0.714 |

**Supplementary Figures:**

*Supplementary Figure 1.* Pie chart showing the proportions of fungal sequence reads across all analysed samples assigned to each of the assessed main functional groups. The majority of sequence reads are classified as saprotrophs or unclassified taxa, whereas only a small proportion of reads are classified as plant pathogens or arbuscular mycorrhizal fungi.


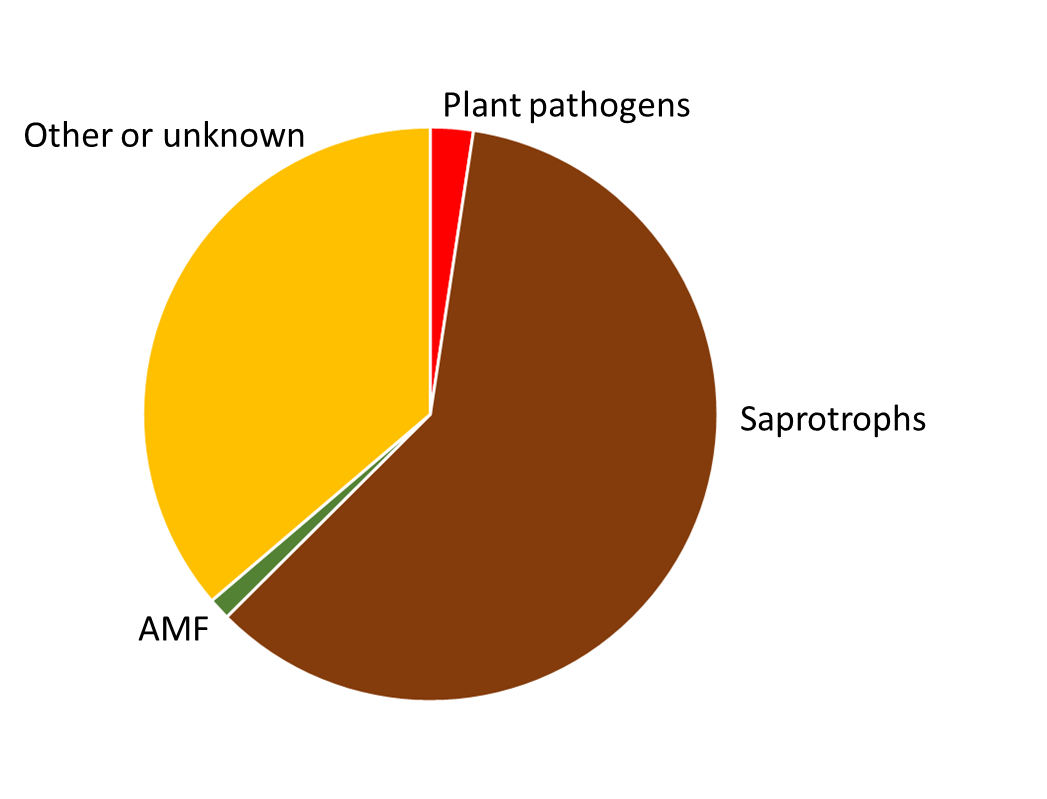


*Supplementary Figure 2.* Forb:grass ratio in the conditioning plant communities created soil legacy effects on grass cover in the responding plant communities that were consistent across all six plant communities. Statistical output of the full statistical model can be found in Supplementary Table 3.

*
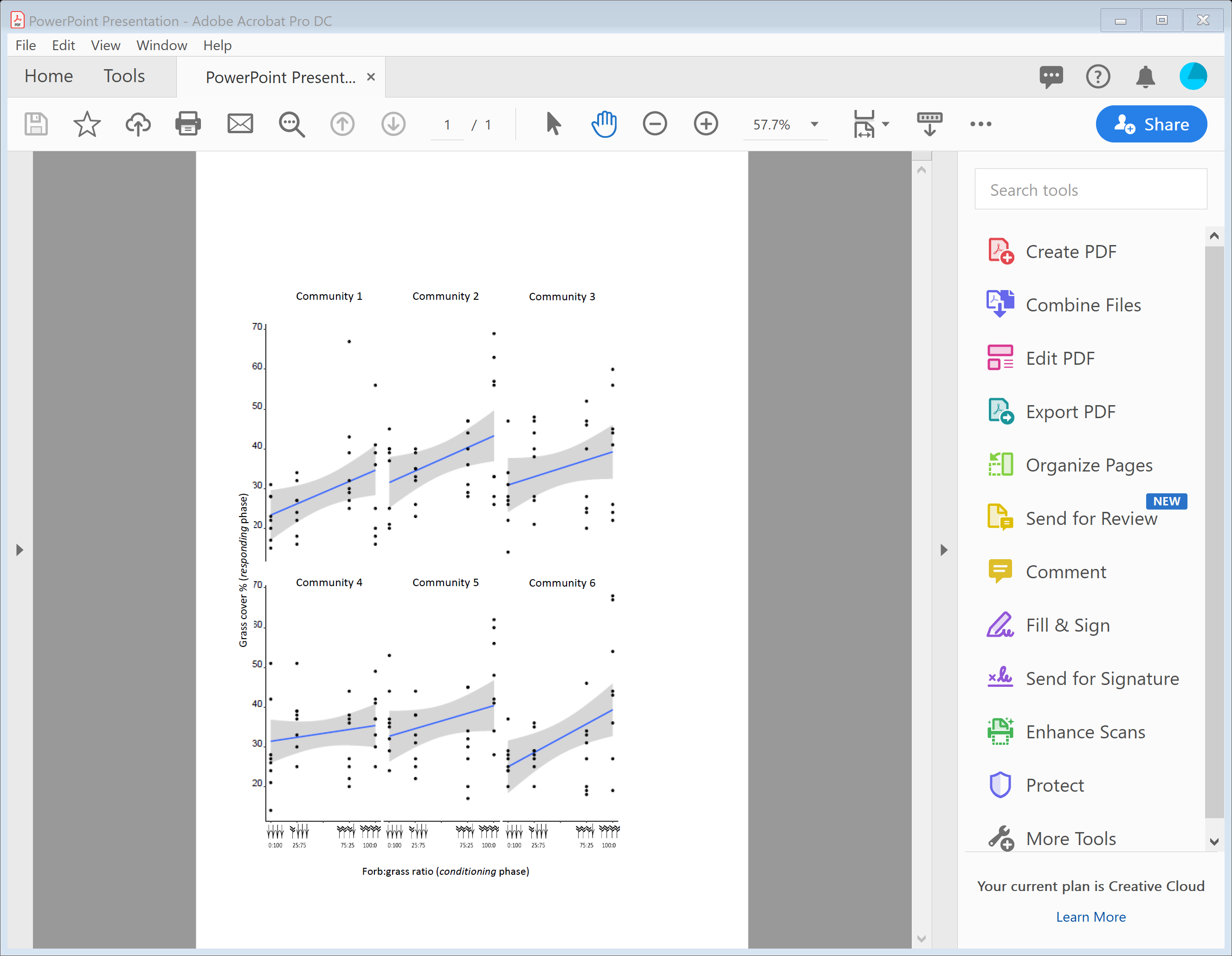
*

*Supplementary Figure 3.* Forb:grass ratio in the conditioning plant communities created soil legacy effects on forb cover in the responding plant communities that were consistent across all six plant communities. Linear trend lines were fit with 95% confidence intervals. Statistical output of the full statistical model can be found in Supplementary Table 3.


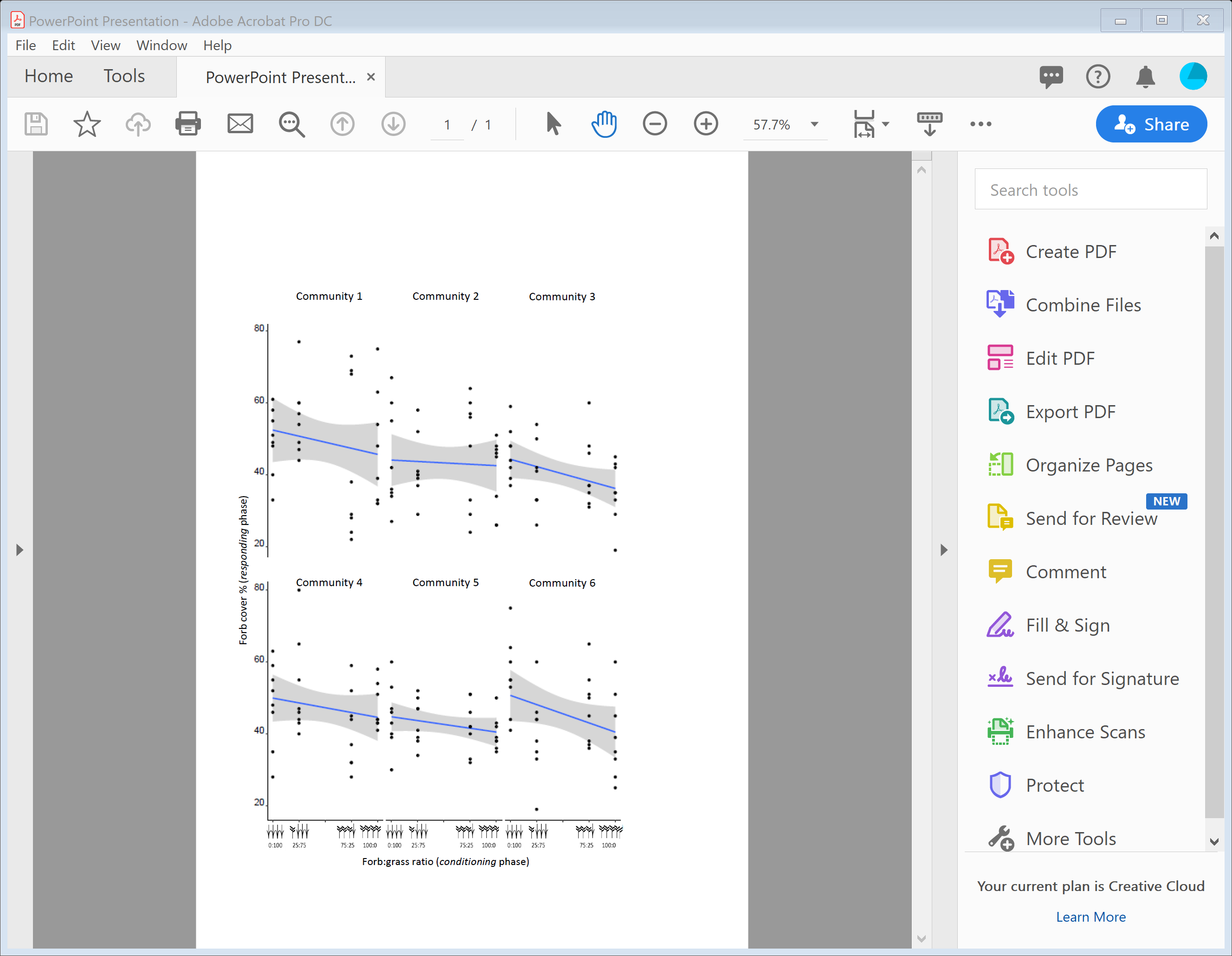


*Supplementary Figure 4:* Correlation plots showing relationships between the relative cover of forbs and grasses at subplot-level in the conditioning phase and the relative cover in the responding phase (August 2018) of the field experiment. Linear trend lines were fit with 95% confidence intervals. Shown are R^2^, F- and p-values for the relationship based on general linear models.


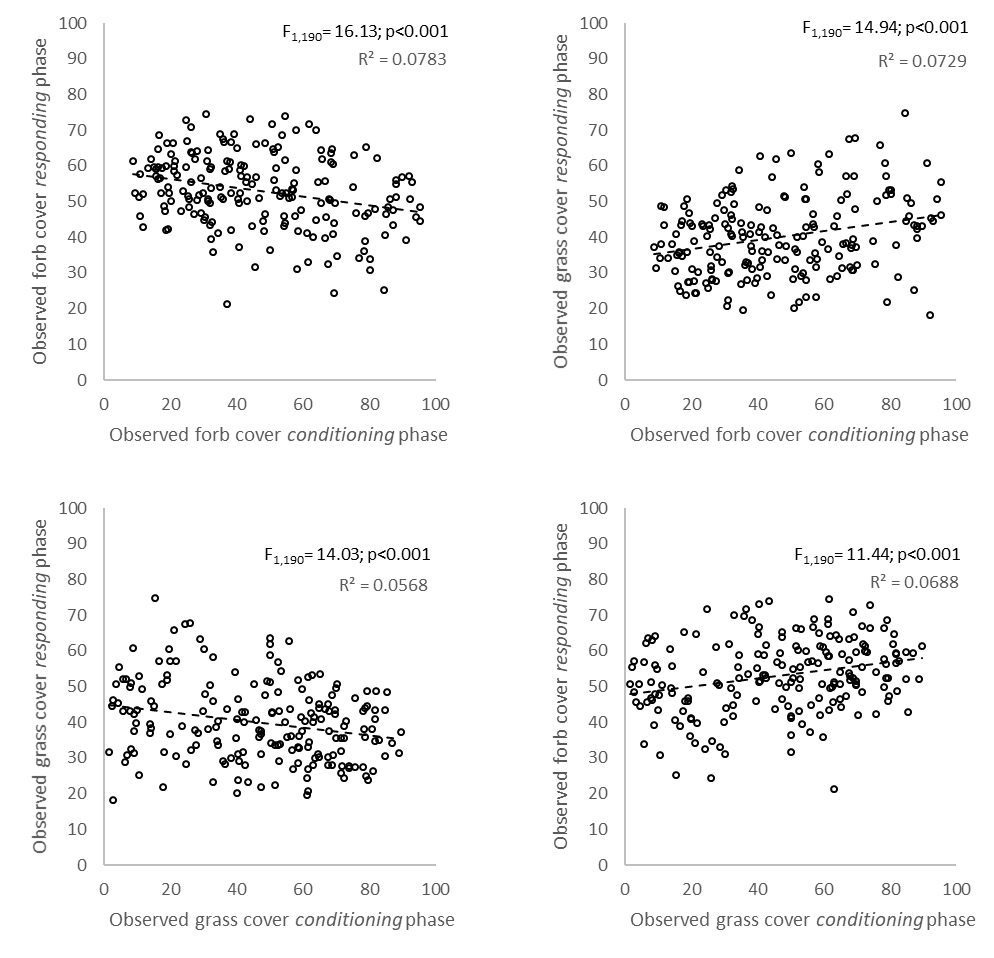


*Supplementary Figure 5.* The effects of conditioning plant community, conditioning time and forb:grass ratio on responding plant community structure. Shown are non-metric multidimensional scaling (NMDS) ordination plots, based on Bray-Curtis dissimilarities. For visualisation purposes, the NMDS plots were faceted by conditioning plant community. Colors from light to dark represent increasing forb:grass ratios. Triangles represent communities in subplots that were conditioned for one year, circles represent communities in subplots that were conditioned for two years. Two-dimensional (k=2) stress value is 0.22 (for the analysis five dimensions were used, k=5, stress is 0.15). Full statistical output can be found in Supplementary Table 4.

 
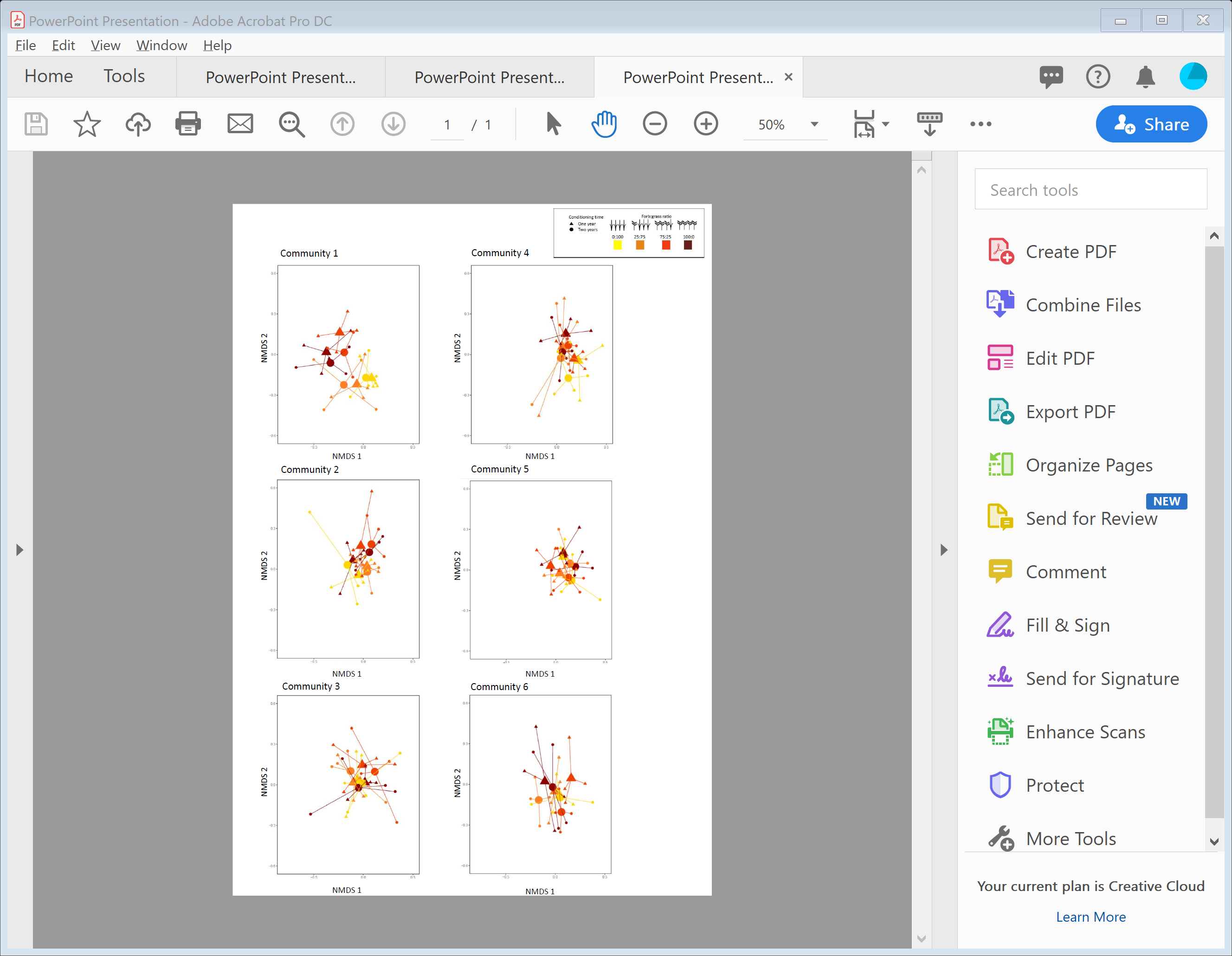


*Supplementary Figure 6.* Biplots showing the output of redundancy analyses (RDA) testing the effect of forb:grass ratio in the conditioning plant community on responding plant community composition. Six separate analyses were performed for each of the six conditioning plant communities. Markers represent the four levels of the forb:grass ratio. Colors from light to dark represent increasing forb:grass ratios. Arrows represent responding plant species.  In the communities where the forb:grass ratio had a significant effect on the responding plant community species, the arrows were coloured for the 24 species from the two species pools (Supplementary Table 1,2), with grasses in light yellow arrows and forbs in dark red arrows. Non-sown species are depicted in dark blue arrows with open arrow heads. Presented are Pseudo-F values and p-values.


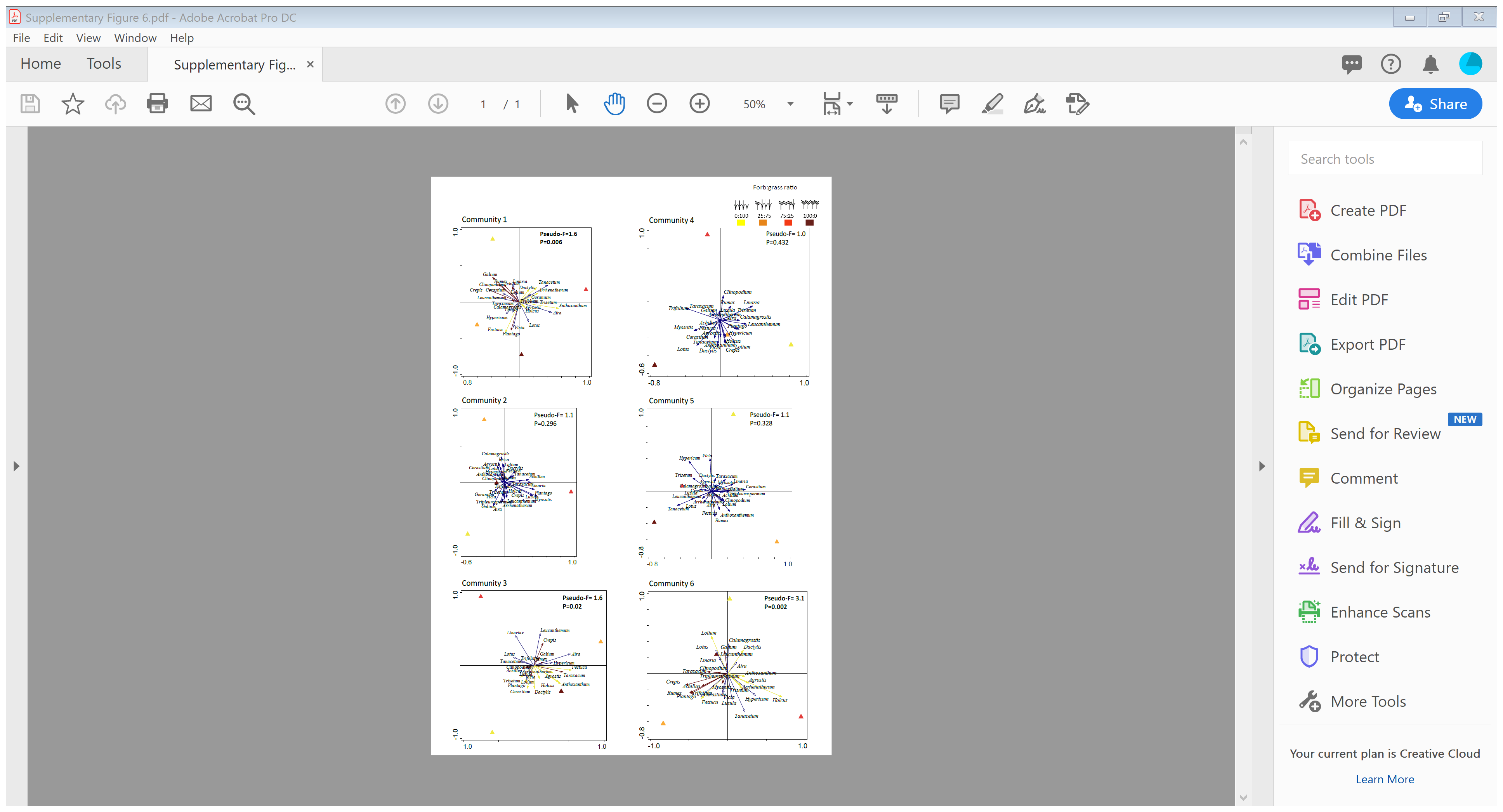


*Supplementary Figure 7.* Correlation plots between individual cover of sown conditioning plant species (Supplementary Table 1) and responding plant species (all plant species present). Correlation plots were made separately for each of the conditioning plant communities to visualize community-specific patterns. In all cases, only those species were included that were present in at least three plots and that had a mean cover of > 0.25% across all of the respective conditioning community plots. Species in black font are conditioning plant species. Species in red font are responding plant species. Correlation plots were corrected for false discovery rates and only those relationships that were significant at p<0.01 are visualized. Red colors indicate a negative relationship and blue colors indicate a positive relationship.


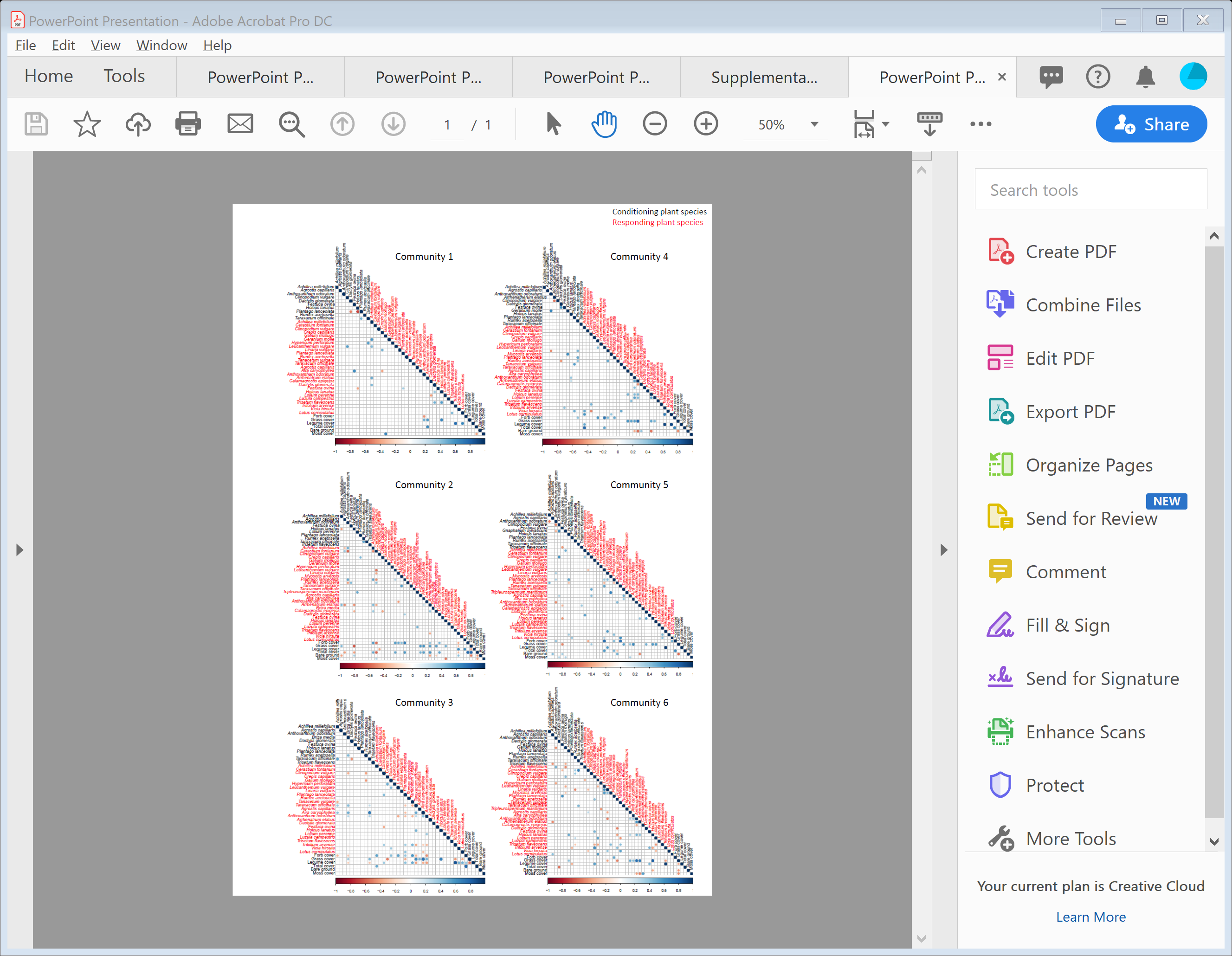


*Supplementary Figure 8.* Forb:grass ratio in the conditioning plant communities affected soil fungal pathogens, but not arbuscular mycorrhizal fungi or saprotrophs. Presented are relative read numbers of total fungal pathogens, grass-specific pathogens, the grass pathogen *Slopeiomyces cylindrosporus*, forb-specific pathogens, arbuscular mycorrhizal fungi and saprotrophs, separated by conditioning plant community. Output of the full statistical model can be found in Supplementary Table 5.


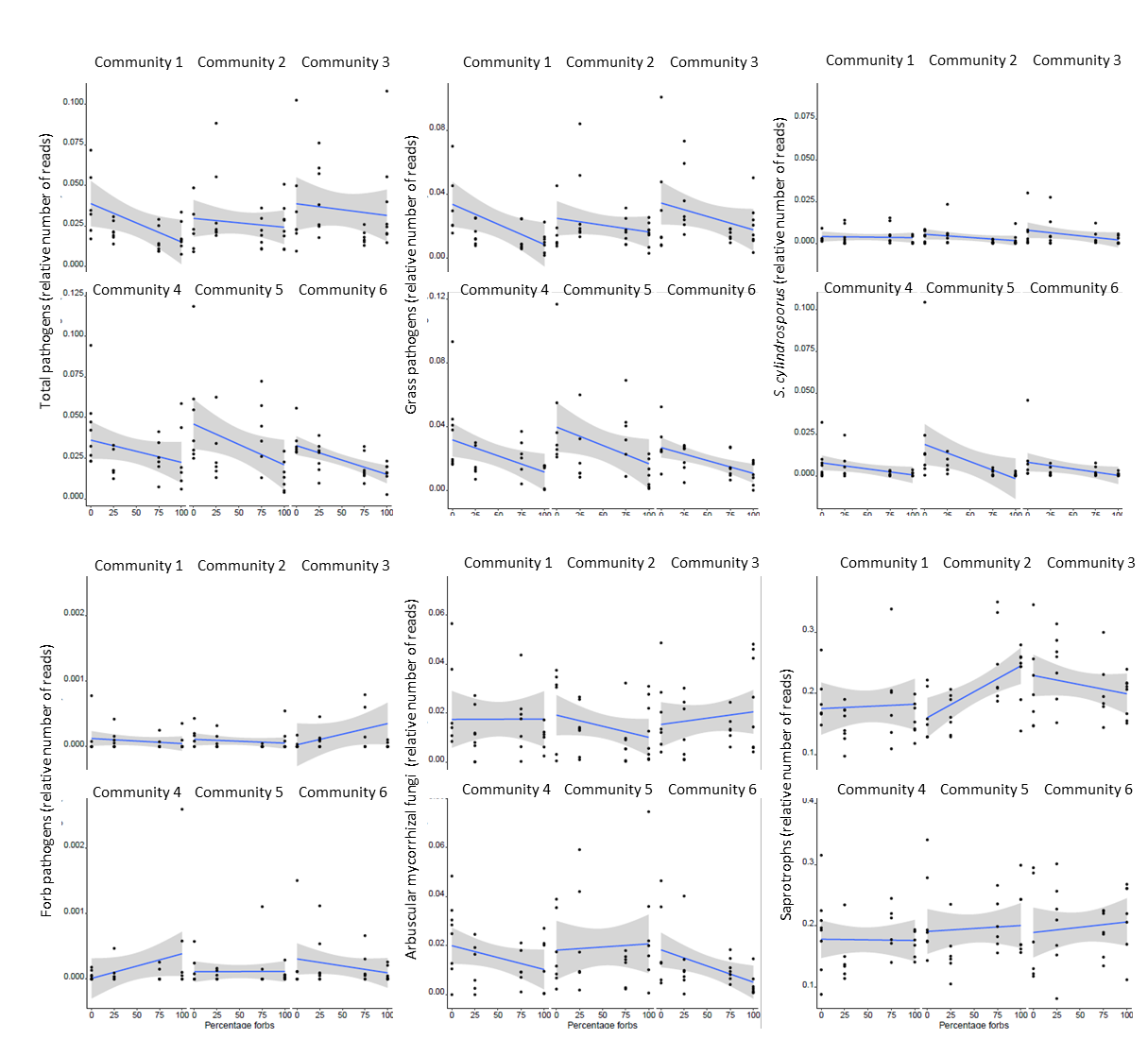


*Supplementary Figure 9.* The effects of conditioning plant community, conditioning time and forb:grass ratio on soil bacterial community structure. Shown are non-metric multidimensional scaling (NMDS) ordination plots, based on Bray-Curtis dissimilarities. For visualisation purposes, the NMDS plots were faceted by conditioning plant community. Colors from light to dark represent increasing forb:grass ratios. Triangles represent communities on plots that were conditioned for one year, circles represent communities on plots that were conditioned for two years. Data filtering is described in the supplementary methods. Two-dimensional (k=2) stress value is 0.18. Full statistical output can be found in Supplementary Table 6.


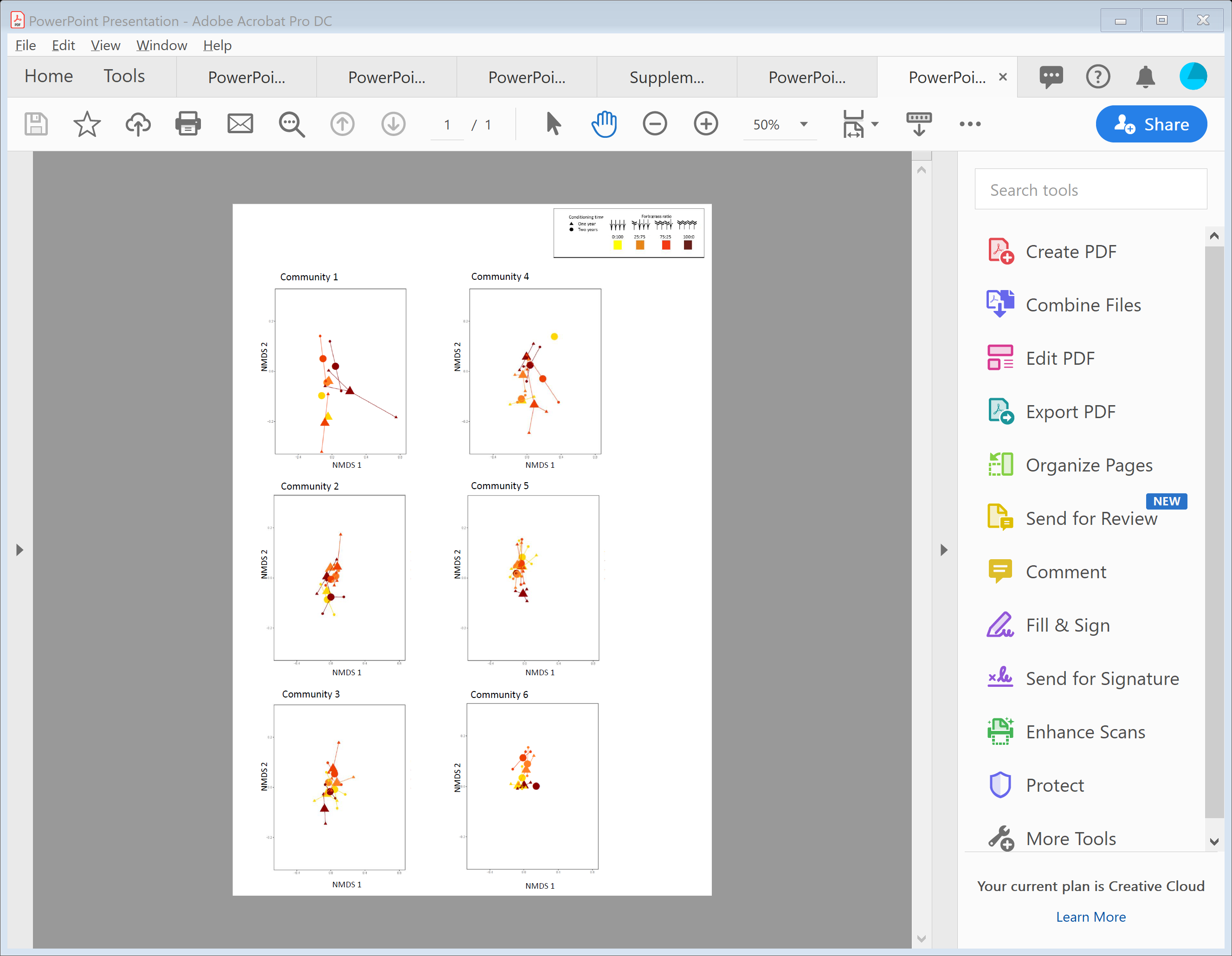


*Supplementary Figure 10.* The effects of conditioning plant community, conditioning time and forb:grass ratio on soil fungal community structure. Shown are non-metric multidimensional scaling (NMDS) ordination plots, based on Bray-Curtis dissimilarities. For visualisation purposes, the NMDS plots were faceted by conditioning plant community. Colors from light to dark represent increasing forb:grass ratios. Triangles represent communities on plots that were conditioned for one year, circles represent communities on plots that were conditioned for two years. Data filtering is described in the supplementary methods. Two-dimensional (k=2) stress value is 0.19. Full statistical output can be found in Supplementary Table 5.


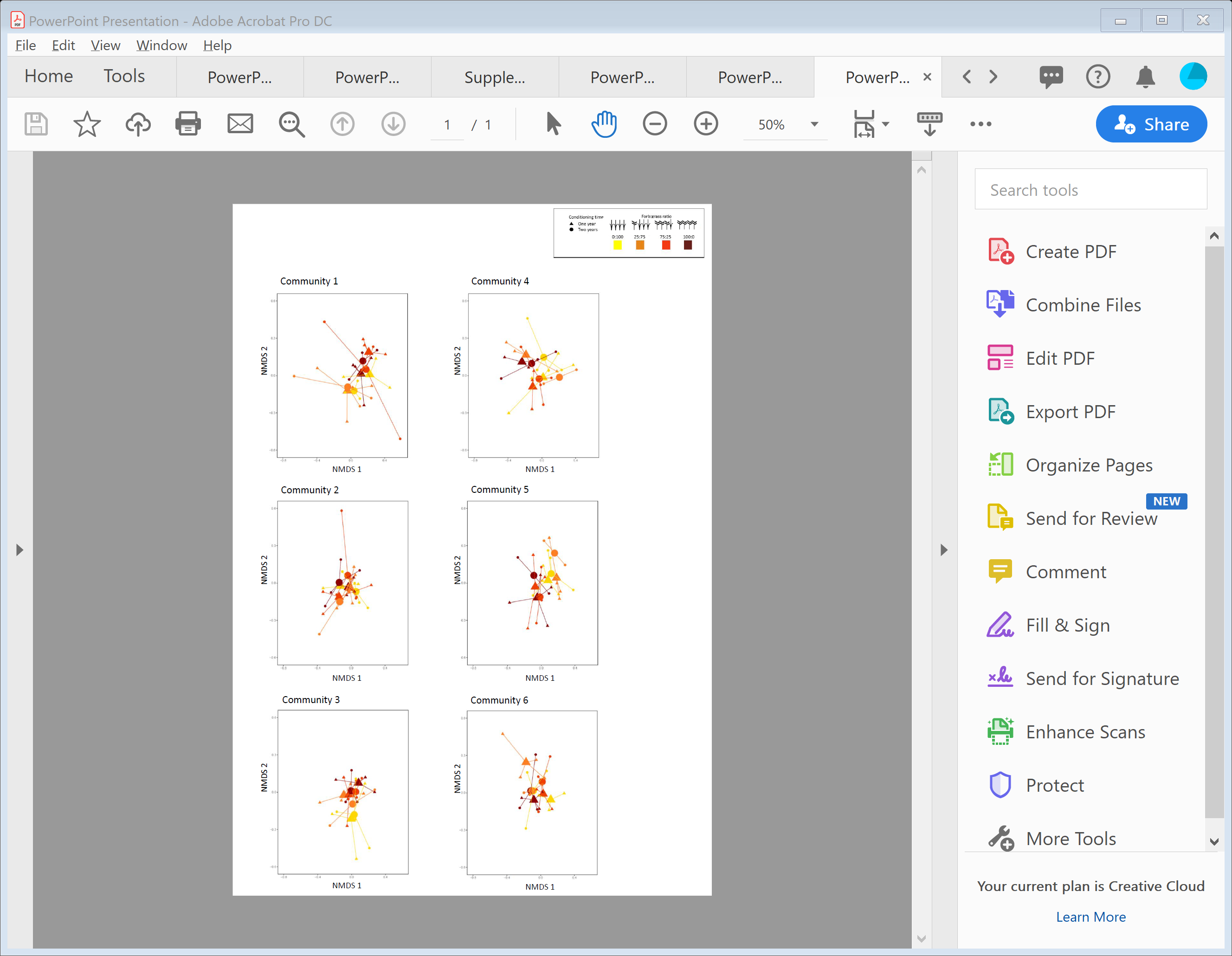

Supplement: Supplementary file 1 — Supplementary Material [file ELE-23-973-s001.docx]
